# Endoplasmic reticulum stress in antitumor immunity and immunotherapy resistance: mechanisms and therapeutic implications

**DOI:** 10.1186/s12943-026-02674-x

**Published:** 2026-04-27

**Authors:** Yingfan Zhao, Yunzhi Shang, Ting Luan, Zhong Yu, Yang Yue, Xing Niu, Hui Li, Shizhuo Wang, Ce Wang, Lu Liu

**Affiliations:** 1 Department of Orthodontics, School and Hospital of Stomatology, China Medical University, Shenyang, Liaoning 110001 China; 2 Department of Neurology, Shengjing Hospital of China Medical University, Shenyang, Liaoning 110004 China; 3 Department of Critical Care Medicine, Shengjing Hospital of China Medical University, Shenyang, Liaoning 110004 China; 4 Department of Obstetrics and Gynecology, Shengjing Hospital of China Medical University, Shenyang, Liaoning 110004 China; 5 China Medical University, Shenyang, Liaoning 110122 China; 6 Department of Gastroenterology, Shengjing Hospital of China Medical University, Shenyang, Liaoning 110004 China; 7 Department of Pediatrics, Shengjing Hospital of China Medical University, Shenyang, Liaoning 110004 China; 8 Department of Preventive Dentistry, School and Hospital of Stomatology, Liaoning Provincial Key Laboratory of Oral Diseases, China Medical University, Shenyang, Liaoning 110001 China

**Keywords:** Endoplasmic reticulum stress, Antitumor immunity, Tumor microenvironment, Immunotherapy resistance, Unfolded protein response

## Abstract

Tumor cells utilize various strategies to enable themselves to survive under adverse conditions and to inhibit the development of antitumor immunity. Factors in the tumor microenvironment (TME), e.g., hypoxia, oxidative stress, and nutrient deprivation, can impair the protein-folding ability of the endoplasmic reticulum (ER) and destroy ER homeostasis both in tumor and immune cells, leading to ER stress. This stress is characterized by the accumulation of misfolded or unfolded proteins. Sensing and responding to ER stress is coordinated by the unfolded protein response (UPR), an integrated signaling pathway controlled by three ER stress sensors: inositol-requiring enzyme 1α (IRE1α), protein kinase R-like ER kinase (PERK), and activating transcription factor 6 (ATF6). In addition to endowing tumor cells with enhanced abilities for tumorigenesis, metastasis, and treatment resistance, aberrant activation of ER stress also impairs antitumor immunity by modulating the phenotype and function of immune cells in the TME. Therapeutic interventions targeting ER stress can achieve direct tumor-killing effects and simultaneously enhance antitumor immune responses. Here we provide a comprehensive overview of the intrinsic and extrinsic mechanisms by which ER stress shapes antitumor immunity and promotes immunotherapy resistance. By understanding these mechanisms, we discuss that ER stress-targeted strategies hold potential to reinvigorate antitumor immunity and improve immunotherapy outcomes. Furthermore, we explore the potential of ER stress as prognostic and predictive biomarkers for cancer immunotherapy. A thorough understanding of how ER stress affects antitumor immunity, as well as how to improve cancer immunotherapy by modulating ER stress is critical for translating these findings into clinical use.

## Background

Immunotherapy has revolutionized the clinical management of a wide range of human malignancies [1]. Regrettably, only a fraction of patients achieve long-term durable tumor control following immunotherapy [1, 2]. Most patients present either primary or acquired resistance to immunotherapy [3]. The resistance to immunotherapy is caused by complex interactions and multiple dynamic mechanisms in the tumor microenvironment (TME) [4]. Accordingly, identifying biomarkers for predicting immunotherapy response and understanding the mechanisms behind immunotherapy resistance are crucial for enhancing the clinical benefits of immunotherapy.

The endoplasmic reticulum (ER) is a complex and dynamic organelle that modulates numerous cellular pathways, e.g., protein synthesis, protein quality control, and lipid metabolism [5]. Extensive cellular disorders destroy the efficiency of protein folding within the ER and cause the accumulation of misfolded or unfolded proteins within this organelle, a state referred to as “ER stress” [6]. This stress is controlled by the unfolded protein response (UPR), an integrated adaptive pathway [7]. If ER stress is prolonged, the UPR cannot restore cellular homeostasis. Instead, it shifts to a pro-apoptotic response rather than an adaptive response. The oncogenic role of the UPR signaling was first described in 2004 [8] and is now widely accepted. The three sensors, inositol-requiring enzyme 1α (IRE1α), protein kinase R-like ER kinase (PERK), and activating transcription factor 6 (ATF6), are crucial for the adaptation of tumor cells to their changing TME [9]. Accordingly, the chronic activation of ER stress supports the major hallmarks of cancer through favoring autonomous and non-autonomous processes of tumor cells, contributing to the pro-tumorigenic and immunosuppressive TME [10].

Emerging evidence suggests that aberrant activation of ER stress impairs antitumor immunity and contributes to immunotherapy resistance by regulating tumor cell-intrinsic mechanisms (including impairing antigen presentation [11, 12], upregulating immune checkpoint ligands [13, 14], producing immunosuppressive factors [15–17], and downregulating interferon (IFN)-γ signaling [18]) and extrinsic immune mechanisms (including inducing macrophages [19–22], neutrophils [23, 24], and B cells [25] to a pro-tumorigenic and immunosuppressive phenotype, enhancing the immunosuppressive function of myeloid-derived suppressor cells (MDSCs) [26, 27], impairing antigen cross-presentation of dendritic cells (DCs) [11, 28], promoting T cell dysfunction and exhaustion [29–31], and affecting tumor killing of natural killer (NK) cells [32]). Therefore, understanding the molecular mechanisms by which ER stress affects antitumor immunity is crucial for revealing its role in immunotherapy response and resistance. The idea of manipulating ER stress to improve antitumor immunity offers an innovative approach for cancer immunotherapy. It has been demonstrated that therapeutic strategies targeting ER stress pathways can enhance the efficacy of immunotherapy [33–35]. At current, targeting ER stress pathways in cancer immunotherapy is a promising yet challenging frontier. This requires a detailed understanding of how alterations in ER stress pathways affect immunotherapy efficacy, as well as how these pathways interact with the TME.

In this review, we focus on the intrinsic and extrinsic mechanisms by which ER stress affects antitumor immunity and immunotherapy resistance rather than cancer progression in general. Furthermore, we explore the potential of ER stress-targeted strategies in enhancing immunotherapy efficacy and overcoming resistance, especially in combination with immune checkpoint inhibitors (ICIs). Finally, we discuss the clinical significance of ER stress as prognostic and predictive biomarkers for cancer immunotherapy.

## Overview of ER stress signaling

**Fig. 1:**
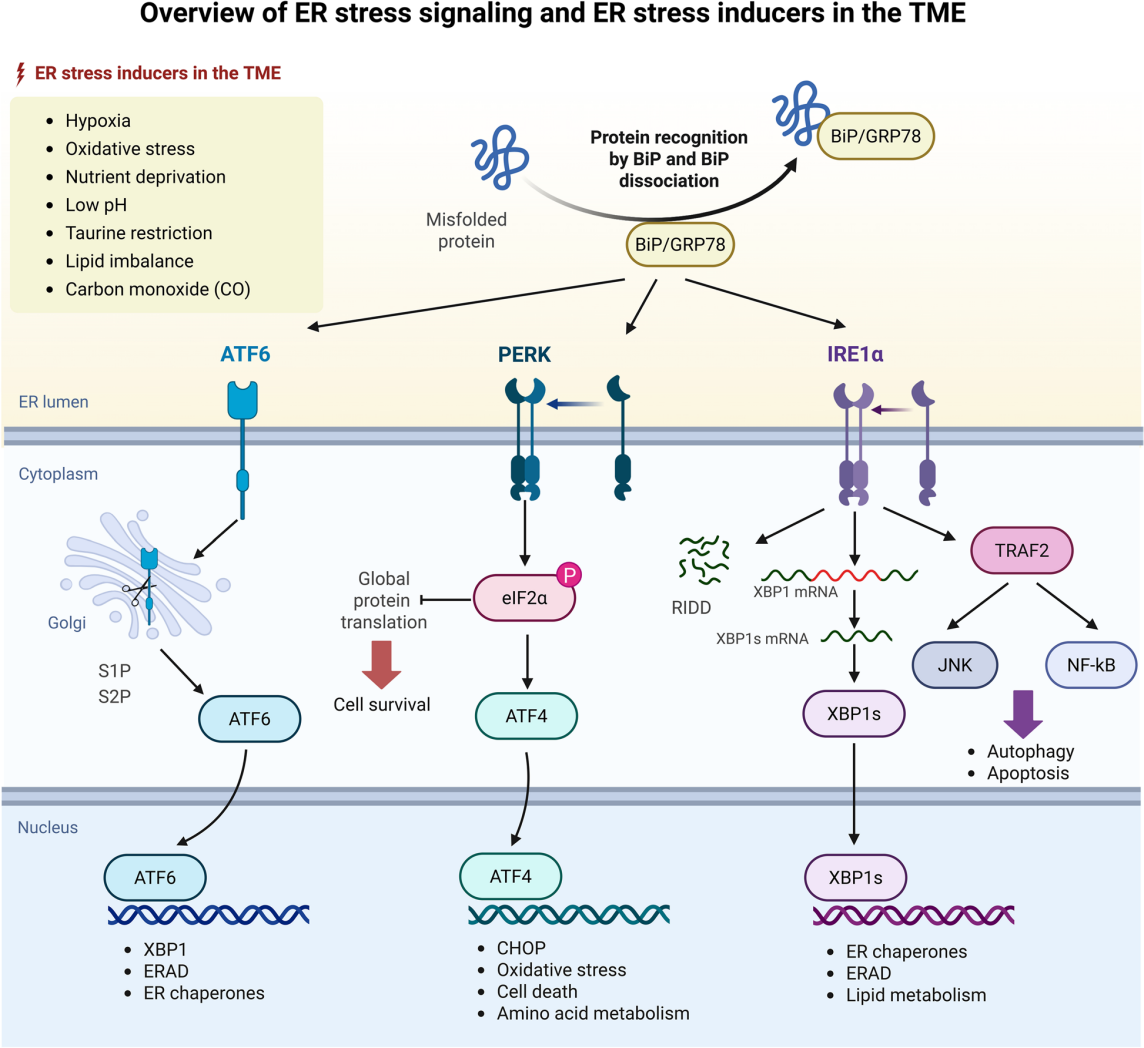
Overview of ER stress signaling and ER stress inducers in the TME. The UPR is controlled by three major ER-resident sensors: IRE1α, PERK, and ATF6. The mechanisms involved in sensing stress by the three sensors are governed by the ER chaperone BiP/GRP78. IRE1α has kinase activity and endoribonuclease (RNase) activity within the cytoplasmic domain. In response to ER stress, the kinase activity of IRE1α induces its autogenous transphosphorylation, thereby activating the RNase activity of IRE1α. IRE1α induces the generation of an active transcription factor XBP1s and degrades specific mRNAs and/or microRNAs via RIDD. Moreover, IRE1α induces autophagy and apoptosis by interacting with TRAF2, activating the JNK and NF-kB signaling. Similar to IRE1α, PERK undergoes the dimer and trans-autophosphorylation in response to ER stress. Subsequently, eIF2α is phosphorylated, which causes overall protein synthesis degradation and preferentially induces ATF4 and CHOP. The protein synthesis degradation promotes cell survival. In contrast, transcriptional regulation by ATF4 and CHOP enhances protein synthesis, causing oxidative stress and cell death. In response to ER stress, ATF6 exports from the ER and undergoes transmembrane cleavage via the proteases S1P and S2P, two Golgi-resident proteases. The released cytoplasmic ATF6 fragment is an active transcription factor, which upregulates a group of UPR-associated genes. As a main ER chaperone, GRP78 plays a key role in folding and processing of nascent membrane and secretory proteins, which is essential for activating the three major sensors. Several factors in the TME induce ER stress, such as hypoxia, oxidative stress, nutrient deprivation, low pH, taurine restriction, lipid imbalance, and carbon monoxide (CO). Abbreviations: ER, endoplasmic reticulum; TME, tumor microenvironment; IRE1α, inositol-requiring enzyme 1α; PERK, protein kinase R-like ER kinase; ATF6, activating transcription factor 6; BiP, binding-immunoglobulin protein; GRP78, glucose‐regulated protein 78; RIDD, regulated IRE1-dependent decay; XBP1s, spliced form of X-box-binding protein 1; TRAF2, tumor necrosis factor receptor-associated factor 2; eIF2α, eukaryotic translation initiation factor 2 α; ATF4, activating transcription factor 4; CHOP, C/EBP homologous protein; ERAD, ER-associated degradation

Most secreted and cell-surface proteins can be targeted into the ER for protein-folding and maturation in eukaryotic cells. When the load of misfolded or unfolded proteins within this organelle is excessive, the UPR is triggered to alleviate ER stress. The UPR is primarily controlled by three major ER-resident sensors: IRE1α, PERK, and ATF6 (Fig. 1) [36]. These sensors are interconnected and together produce a coordinated response. The mechanisms involved in sensing stress by the three sensors are governed by the ER chaperone binding-immunoglobulin protein (BiP; also called glucose‐regulated protein 78 (GRP78)).

### ER-resident sensors and chaperones

#### IRE1α

As the most evolutionally conserved ER stress sensor, IRE1α is a type I transmembrane protein with kinase activity and endoribonuclease (RNase) activity within the cytoplasmic domain.

The kinase activity of IRE1α mediates its autogenous transphosphorylation in response to ER stress-mediated IRE1α dimerization, thereby activating the RNase activity of IRE1α. Most IRE1α signaling outputs are associated with the RNase activity. In response to ER stress, IRE1α removes a 26-nucleotide fragment from XBP1 mRNA, generating an active transcription factor XBP1s [37]. Moreover, IRE1α RNase degrades specific mRNAs and/or microRNAs through regulated IRE1-dependent decay (RIDD) [38, 39].

In addition to its catalytic activity, IRE1α has scaffold functions related to cell migration, calcium signal transduction, and bioenergetics [40, 41]. Furthermore, IRE1α induces autophagy and apoptosis by interacting with tumor necrosis factor (TNF) receptor-associated factor (TRAF)2, thereby activating the JNK and NF-kB signaling [42–44]. The IRE1α signaling has been widely explored in solid and hematological tumors [11, 29, 45, 46].

#### PERK

PERK is a type I transmembrane ER-resident protein [47]. Similar to IRE1α, PERK undergoes the dimer and trans-autophosphorylation in response to ER stress, causing the phosphorylation of eukaryotic translation initiation factor 2 α (eIF2α) [48]. This event causes overall protein synthesis degradation, preferentially translates activating transcription factor 4 (ATF4), and induces C/EBP homologous protein (CHOP) [49]. The protein synthesis degradation, induced by eIF2α phosphorylation, rather than ATF4 mRNA translation, promotes cell survival [50]. In contrast, transcriptional regulation by ATF4 and CHOP enhances protein synthesis, causing oxidative stress and cell death.

Given the dual role of eIF2α phosphorylation, this process needs to be precisely regulated, ensuring protein-folding homeostasis in the ER. After stimulation, eIF2α undergoes transient phosphorylation, which acutely and transiently inhibits protein synthesis. Subsequently, induction of ATF4 and CHOP and their downstream targets is implicated in restoring protein synthesis. When eIF2α is phosphorylated, ATF4 and CHOP mRNAs are preferentially translated [51–53].

During ER stress, there is a negative feedback loop: eIF2α-mediated protein synthesis inhibition induces GADD34 expression, and GADD34 dephosphorylates eIF2α, promoting translational recovery [54, 55]. If protein synthesis increases before the restoration of protein homeostasis, reactive oxygen species (ROS) are produced, a necessary signal for cell apoptosis. A recent study found that GADD34 regulates the IRE1α-XBP1 signaling by directly interacting with IRE1α [56], independently of its canonical role in eIF2α dephosphorylation [54, 55]. Pharmacological suppression of GADD34 using Sephin1 suppresses XBP1 splicing and alleviates CAR-T cell exhaustion, enhancing tumor-killing capacity [56].

In response to ER stress, the interaction between PERK and mitochondrial ATAD3A increases, thereby regulating local translation by competing with eIF2α binding [57]. Moreover, the binding attenuates the PERK signaling and rescues the expression of mitochondrial proteins. QRICH1 is an effector of the PERK/eIF2α signaling, dictating cell fate under ER stress via transcriptionally controlling proteostasis [58]. Signal recognition particle 14 (SRP14)-driven elongation arrest is crucial for protein co-translational translocation [59]. SRP14 expression is reduced upon ER stress, which requires PERK-induced eIF2α phosphorylation [60]. This process is controlled by ubiquitination and proteasomal degradation [60]. The PERK-SRP14 signaling-mediated translocational attenuation is a protective UPR mechanism in alleviating ER stress. In addition, PERK possesses lipid kinase activity, which catalyzes and converts diacylglycerol to phosphatidic acid [61].

At current, the roles and cellular and molecular mechanisms of the PERK signaling in tumor initiation and progression have been extensively studied [62–64].

#### ATF6

ATF6 is a type II ER transmembrane protein with an N-terminal cytoplasmic b-ZIP domain [65]. In response to ER stress, ATF6 exports from the ER and undergoes transmembrane cleavage via the proteases S1P and S2P, two Golgi-resident proteases [66, 67]. The released cytoplasmic ATF6 fragment is an active transcription factor, which upregulates UPR-associated genes, including those that enhance the ER-associated degradation (ERAD) signaling [68]. It interacts with XBP1s by forming heterodimers, driving specific gene expression programs [69]. ATF6 also acts as an “off-switch” of the IRE1α signaling [70].

Emerging evidence suggests that chronic activation of ATF6 signaling selectively activates a tumor-promoting microbiota by altering lipid metabolism [71]; facilitates colorectal cancer growth and stemness through Wnt-related programs [72]; and contributes to liver tumor initiation and glycolysis-dependent immunosuppression [73]. However, in contrast to IRE1α and PERK, less is known about ATF6. The roles of ATF6 in tumor initiation, progression, and antitumor immunity deserve further exploration.

#### BiP/GRP78

The chaperone BiP/GRP78 is one of the most abundant proteins in the ER, which contains an ER-directed signal sequence [74]. As a main ER chaperone, GRP78 is implicated in folding and processing of nascent membrane and secretory proteins, which is fundamental for activating the three major sensors [75]. GRP78 is also regarded as a direct ER stress sensor that causes UPR activation. ER stress facilitates the process by which ER chaperones escape from the ER compartment and relocalize to cellular compartments (e.g., nucleus, mitochondria, and cell surface), where they impact cell signaling, proliferation, and survival [10].

In response to ER stress, IRE1α forms a complex with SRC, causing a feed-forward mechanism that induces GRP78 to the cell surface, where CD109 acts as a GRP78-binding partner to block the TGF-β signaling [76]. ATF6 and XBP1s transcriptionally upregulate the ER response gene SHQ1. The GRP78-SHQ1 interaction induces the release of IRE1α, PERK, and ATF6, causing UPR hyperactivation [77]. GRP78 binds to IRE1α and PERK via its NBD, and its release depends on the binding of misfolded proteins to GRP78 SBD. The IRE1α-PERK interaction converts GRP78 from chaperone to ER stress sensor via hindering the binding of its co-chaperones, while losing the stimulation of ATPase [78]. GRP78 also binds to IRE1α via SBD to inhibit the UPR signaling [79]. Aberrant expression of GRP78 participates in nearly all aspects of tumor initiation and progression [80–83].

### Factors in the TME induces ER stress

Hostile factors in the TME elicit ER stress, e.g., hypoxic condition, oxidative stress, nutrient deprivation, low pH, taurine restriction, lipid imbalance, and carbon monoxide (CO) (Fig. 1). Below we discuss the impact of these factors on fostering ER stress.

#### Hypoxia

Hypoxia is a common characteristic of solid tumors, which is associated with resistance to cancer therapies, including immunotherapy [84–86]. In the TME, tumor cells experience hypoxia, causing the accumulation of misfolded or unfolded proteins largely within the ER. Furthermore, hypoxic cells exhibit reduced desaturated lipids, limiting ER expansion and triggering ER stress [87]. Consequently, hypoxia elicits ER stress as a cancer adaptive mechanism [88]. However, only extreme hypoxic condition elicits effective ER stress, while modest hypoxic condition (1 ~ 5% O_2_) exerts a minimal effect on UPR activation [89, 90].

#### Oxidative stress

Protein folding highly relies on the redox state of the ER. The accumulation of ROS disrupts ER proteostasis and elicits ER stress through affecting ER-resident calcium signals and inducing the production of lipid peroxidation by-products [11, 91, 92]. Excessive ROS accumulation facilitates PERK-induced stabilization of Nrf2, thereby limiting oxidative damage [93, 94].

#### Nutrient deprivation

Nutrients in the TME originate from serum or a variety of cell types that constitute the tumor [95]. As the ER is a nutrient-sensing organelle [96], glucose [97] and glutamine [98] deprivation activates ER stress. The restriction of glucose or glutamine destroys the hexosamine biosynthetic signaling. This signaling utilizes the two nutrients to produce uridine diphosphate-N-acetylglucosamine (UDP-GlcNAc), an essential component for N-linked glycosylation and protein folding within the ER. Targeted glucose- or glutamine-dependent tumor inhibition is regarded as a long-term strategy for cancer treatment.

#### Low pH

Tumor cells utilize aerobic glycolysis as their dominant metabolic pathway, generating much lactate that causes acidification of the TME [99]. Under low pH conditions, the proton-sensing receptors are activated, which induce IRE1α, PERK, and ATF6 in a variety of cell types [100–102].

#### Taurine restriction

Taurine is a conditionally essential amino acid that exerts paradoxical roles in cancer, either promoting tumor growth or enhancing antitumor immunity [103]. Tumor cells utilize taurine to sustain metabolism and resist stress, while immune cells in the TME depend on taurine to sustain their effector functions [104–106]. These distinct effects are determined by cell types and local environmental factors.

Taurine sustains ER proteostasis and protein-folding integrity and inhibits ER stress [106]. Additionally, taurine is crucial for conjugating primary bile acids and promoting fat digestion and absorption. Unconjugated or primary bile acids and secondary bile acids elicit ER stress in cancer cells through destroying the ER membrane and enhancing the accumulation of misfolded proteins in this organelle [107]. Upon conjugation with taurine, primary bile acids cannot induce ER stress and reduce the survival-promoting adaptability of tumor cells in the TME [107].

Different bile acids have distinct roles in regulating ER stress responses in CD8^+^ T cells in the TME. Primary bile acids elicit oxidative stress in liver cancer, while the secondary bile acid (lithocholic acid) inhibits T cell function via ER stress [108]. Moreover, taurine-conjugated bile acids and ursodeoxycholic acid alleviate harmful ER stress in T cells and thus preserve their functionality [108].

#### Lipid imbalance

Lipid imbalance in the TME induces ER stress. Cholesterol accumulation within the ER membrane affects the fluidity and ER-resident enzymes and protein chaperones, thereby affecting ER stress [109].

As rapidly proliferating cells, tumor cells require much cholesterol to support membrane biogenesis [110]. Tumor cells respond to hostile extrinsic environment via cholesterol biosynthesis, achieving cellular adaptation and better survival. Inhibition of de novo cholesterol synthesis can activate ER stress-induced tumor cell apoptosis [111]. Furthermore, decreasing extracellular cholesterol by statins provokes severe ER stress and thus elicits antitumor immunity in KRAS-mutant tumors [112].

Similar to tumor cells, activated T cells also proliferate rapidly, so they rely on enhanced cholesterol metabolism to obtain sufficient cholesterol. However, increased cholesterol in the TME activates XBP1 in CD8^+^ T cells, causing immune checkpoint upregulation and CD8^+^ T cell exhaustion [46]. Increased intrinsic cholesterol biosynthesis and uptake is beneficial, while excessive or insufficient exogenous cholesterol leads to T cell dysfunction [113].

Glucosylceramide, a type of sphingolipids, is mainly produced by tumor cells [114]. Altered glucosylceramide levels within the TME elicit ER stress in immune cells [115]. The accumulation of glucosylceramide is induced by aberrant lipid metabolism and elevated glucosylceramide synthase activity. Lipids produced by tumor cells, e.g., β-glucosylceramide, simultaneously trigger macrophage polarization and survival in the TME by inducing ER stress [115].

#### Carbon monoxide (CO)

CO acts as an endogenous gaseous molecule and a neurotransmitter that regulates many cellular functions and the TME [116]. CO levels are usually increased upon heme oxygenase-1 (HO-1) upregulation. This is caused by oxidative stress via the activation of Nrf2 that transcriptionally activates HO-1 under ROS and hypoxic pathways [117]. Elevated CO destroys mitochondrial respiration via interacting with cytochrome c oxidase, causing ROS accumulation, aberrant protein folding, and ER stress [118].

CO exerts different effects on different cell types and metabolic states. In tumor cells, CO treatment promotes cell apoptosis by inducing metabolic stress and mitochondrial dysfunction [119]. Oppositely, low-dose CO exposure induces transient activation of ER stress by activating the PERK signaling in T cells [120]. This elicits mitochondrial biogenesis and autophagy as a protective mechanism in T cells, which depends on the PERK signaling activation. Moreover, autophagy activation induces metabolic and epigenetic reprogramming of T cells, thereby improving their antitumor immunity [120]. More studies are needed to elucidate the specificity of cell types and dose-dependent roles.

## Tumor cell-intrinsic ER stress programs in antitumor immunity and immunotherapy resistance

**Fig. 2:**
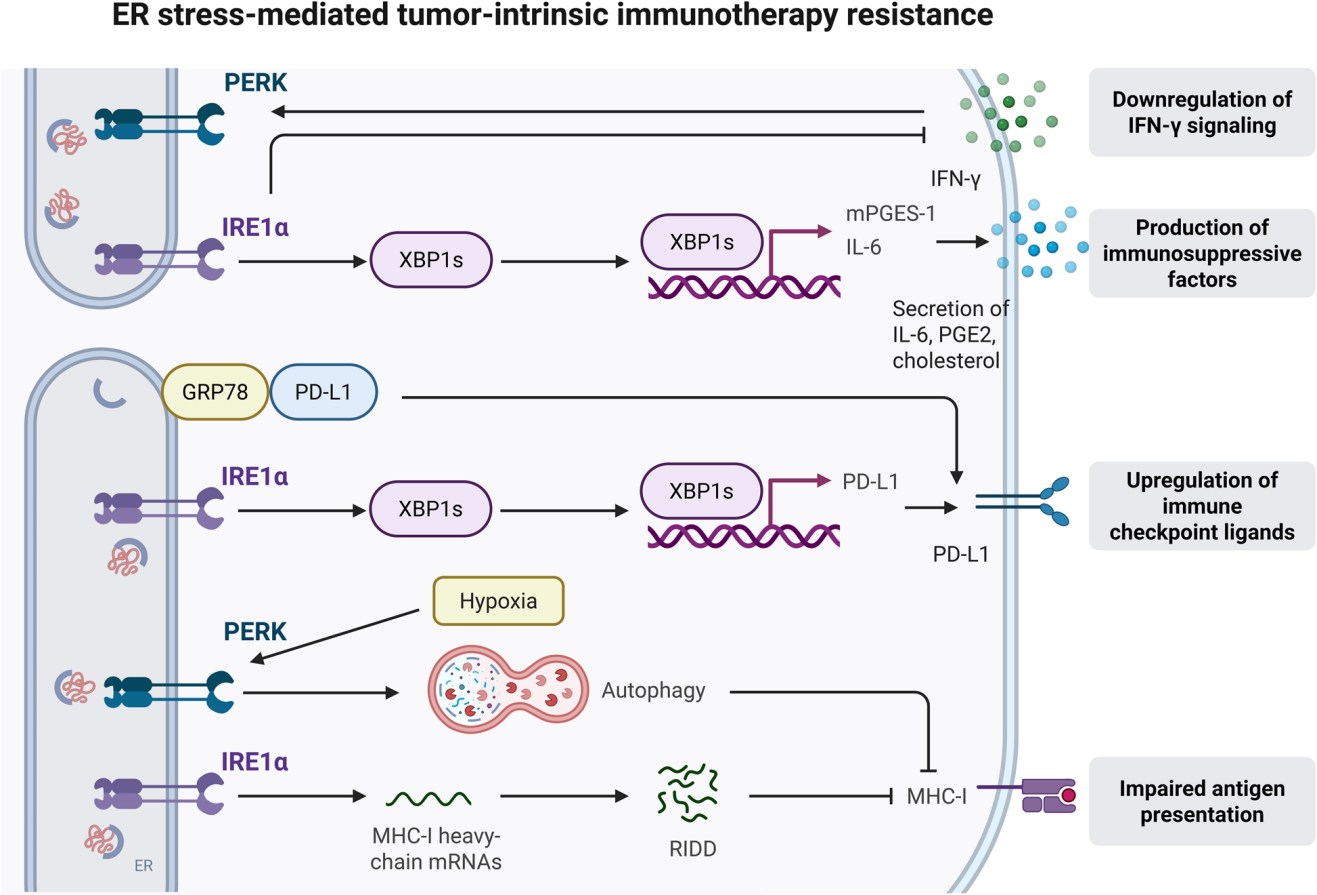
Immunoregulatory effects of tumor cell-intrinsic ER stress. ER stress impairs antigen presentation, upregulates immune checkpoint ligands, induces the production of immunosuppressive factors, and downregulates interferon (IFN)-γ signaling in tumor cells, contributing to tumor immune evasion. The expression of MHC-I is decreased by (i) IRE1α-mediated degradation of MHC-I heavy-chain mRNAs via the RIDD mechanism and (ii) hypoxia-induced autophagy via the PERK signaling. The upregulation of PD-L1 is induced by (i) transcriptional activation mediated by the IRE1α-XBP1 signaling and (ii) enhanced stability PD-L1 protein induced by GRP78. The IRE1α-XBP1 signaling enhances the transcription of immunosuppressive factors (such as IL-6 and PGE2), and promotes the synthesis and secretion of cholesterol. Tumor-intrinsic loss of IRE1α enhances the IFN-γ signaling response by upregulating the IFN-γ transcriptional response genes. Abbreviations: ER, endoplasmic reticulum; IRE1α, inositol-requiring enzyme 1α; PERK, protein kinase R-like ER kinase; GRP78, glucose‐regulated protein 78; RIDD, regulated IRE1-dependent decay; XBP1s, spliced form of X-box-binding protein 1; MHC-I, MHC class I; PD-L1, programmed death ligand 1; IL, interleukin; PGE2, prostaglandin E_2_; mPGES-1, microsomal prostaglandin E synthase-1; IFN, interferon

In addition to directly affecting tumor cell survival and fate [10], tumor cell-intrinsic ER stress also exerts immunoregulatory effects, such as antigen presentation defects, upregulation of immune checkpoint ligands, and immunosuppression. In this section, we summarize the immunoregulatory effects and mechanisms of tumor cell-intrinsic ER stress in antitumor immunity and immunotherapy resistance (Fig. 2). In contrast to IRE1α and PERK, the current research on the impact of tumor cell-intrinsic ATF6 signaling on antitumor immunity and immunotherapy resistance is still relatively limited.

### Impaired antigen presentation

Deficiency in MHC class I (MHC-I) antigen presentation in tumors represents a common mechanism of adaptive immune evasion and immunotherapy resistance [121]. Enhancing MHC-I expression can improve antitumor immunity [122]. The loading of antigenic peptides onto MHC-I relies on the peptide loading complex at the ER, and MHC-I antigen presentation is regulated by the ERAD signaling [123].

The IRE1α signaling activation removes MHC-I heavy-chain mRNAs via the RIDD mechanism, thereby blocking the cross-presentation of antigens [34]. Hypoxia reduces the expression of MHC-I by activating autophagy depending on the PERK signaling, promoting tumor immune escape [12]. Understanding the ER stress-mediated immunoregulatory mechanisms of antigen presentation is crucial for achieving effective immunotherapy.

### Upregulation of immune checkpoint ligands

ER stress upregulates immune checkpoint ligands in cancer cells. The upregulation of programmed death ligand 1 (PD-L1) is an adaptive mechanism controlling immunotherapy resistance [124]. The IRE1α-XBP1 signaling activation prevents CD8^+^ T cell-dependent immune surveillance by increasing PD-L1 transcription [14]. GRP78 interacts with PD-L1 at the ER region and upregulates PD-L1 expression by enhancing its stability in triple-negative breast cancer (TNBC) cells [13]. ER stress also promotes the secretion of exosomal PD-L1 from tumor cells (such as oral squamous cell carcinoma (OSCC) cells [125] and breast cancer cells [126]) and induces M2 macrophage polarization via PD-L1 upregulation in macrophages.

### Production of immunosuppressive factors

To escape from immunosurveillance, tumor cells secrete several immunosuppressive factors (e.g., interleukin (IL)−6/−8 and prostaglandin E_2_ (PGE2)). Tumor cell-intrinsic IRE1α signaling suppresses protective immunity in lung cancer [16]. The intrinsic activation of this signaling in lung cancer cells increases the transcription and secretion of IL-6 [17], and promotes the generation of PGE2 by sustaining microsomal prostaglandin E synthase-1 (mPGES-1) expression [16]. Inhibition of tumor cell-intrinsic IRE1α signaling can delay tumor growth, improve antitumor immunity, and prolong survival in non-small cell lung cancer (NSCLC) mouse models. Tumor cell-intrinsic XBP1 promotes the synthesis and secretion of cholesterol, which can be internalized by MDSCs, thereby activating MDSCs and suppressing antitumor immunity [15].

### Downregulation of interferon (IFN)-γ signaling

The IFN-γ signaling response is crucial for antitumor immunity, which represents a common feature of tumors responding to immunotherapy. The IFN-γ signaling downregulation causes immunotherapy resistance [127].

Tumor-intrinsic loss of IRE1α boosts the IFN-γ signaling response by upregulating the IFN-γ transcriptional response genes (such as HELZ2, ZBP1, and PML) in prostate cancer [18]. The increase in the IFN-γ signaling response has been observed in many cell types within the TME of prostate cancer, especially antigen-presenting cells [18]. Furthermore, the loss of IRE1α in tumor cells decreases the abundance of immunosuppressive cells (such as TAMs and regulatory T cells (Tregs)) and increases the abundance of CD8^+^ T and NK cells within the TME [18]. This improves antitumor immune responses and causes tumor regression and prolonged survival in tumor-bearing mice of prostate cancer [18]. Another study on a NSCLC mouse model also suggests that tumor-intrinsic loss of IRE1α increases CD8^+^ T cells and decreases Tregs, but without significant effects on TAMs [16]. Therefore, in different cancer types, the influence of the tumor-intrinsic IRE1α signaling on the TME may have both similarities and differences.

In addition to playing a role in activating cellular immunity and enhancing antitumor immune responses, active IFN-γ signaling can also induce apoptosis and cell cycle arrest in cancer cells [128]. Treatment with IFN-γ induces ER stress in lung adenocarcinoma (LUAD) cells via activating the JAK1/2-STAT1 and PI3K-AKT-mTOR signaling pathways [128]. This consequently impairs autophagic flux and induces apoptosis and cell cycle arrest in LUAD cells. When ER stress cannot be reversed and cell function deteriorates, it usually leads to cell death. Therefore, ER stress of tumor cells triggered by IFN-γ is one of the potential mechanisms underlying IFN-γ-mediated antitumor effects.

## Immune-cell-intrinsic effects of ER stress in antitumor immunity and immunotherapy resistance

**Fig. 3:**
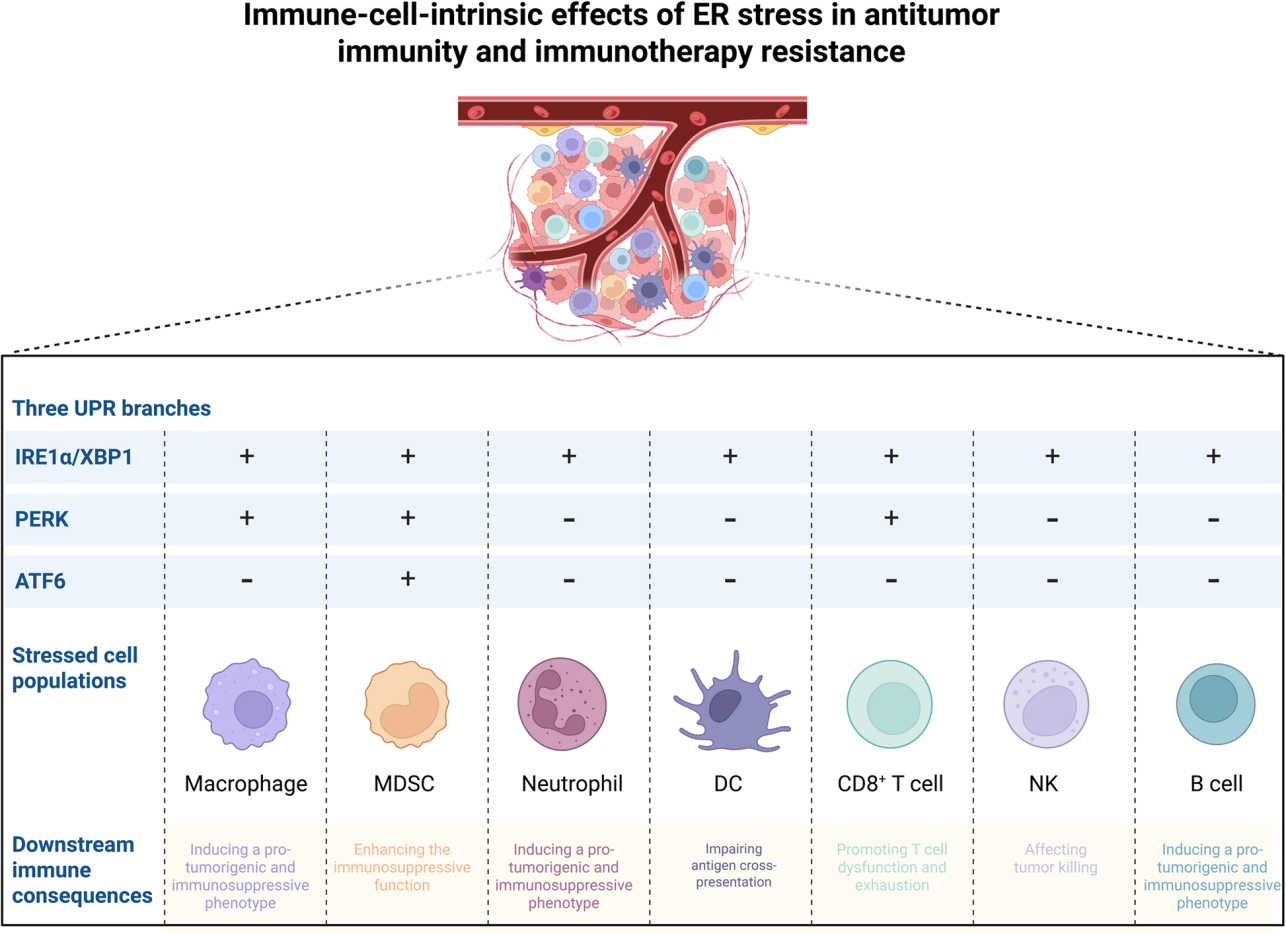
Immune-cell-intrinsic effects of ER stress in antitumor immunity and immunotherapy resistance. Aberrant activation of ER stress sensors (IRE1α/XBP1, PERK, or ATF6) induces macrophages, neutrophils, and B cells to a pro-tumorigenic and immunosuppressive phenotype; enhances the immunosuppressive function of MDSCs; impairs antigen cross-presentation of DCs; promotes T cell dysfunction and exhaustion; and affects tumor killing of NK cells. Abbreviations: ER, endoplasmic reticulum; UPR, unfolded protein response; IRE1α, inositol-requiring enzyme 1α; XBP1, X-box-binding protein 1; PERK, protein kinase R-like ER kinase; ATF6, activating transcription factor 6; DC, dendritic cell; MDSC, myeloid-derived suppressor cell; NK, natural killer

Cancer immunosurveillance requires the collaborative action of the innate and adaptive immune systems to eliminate tumors [129–131]. ER stress regulates the phenotype and function of tumor-infiltrating immune cells, thereby affecting antitumor immune responses and immunotherapy efficacy (Fig. 3). Unlike IRE1α and PERK, the current research on the influence of the ATF6 signaling in immune cells within the TME remains weak.

### Myeloid rewiring

#### Macrophages

**Fig. 4:**
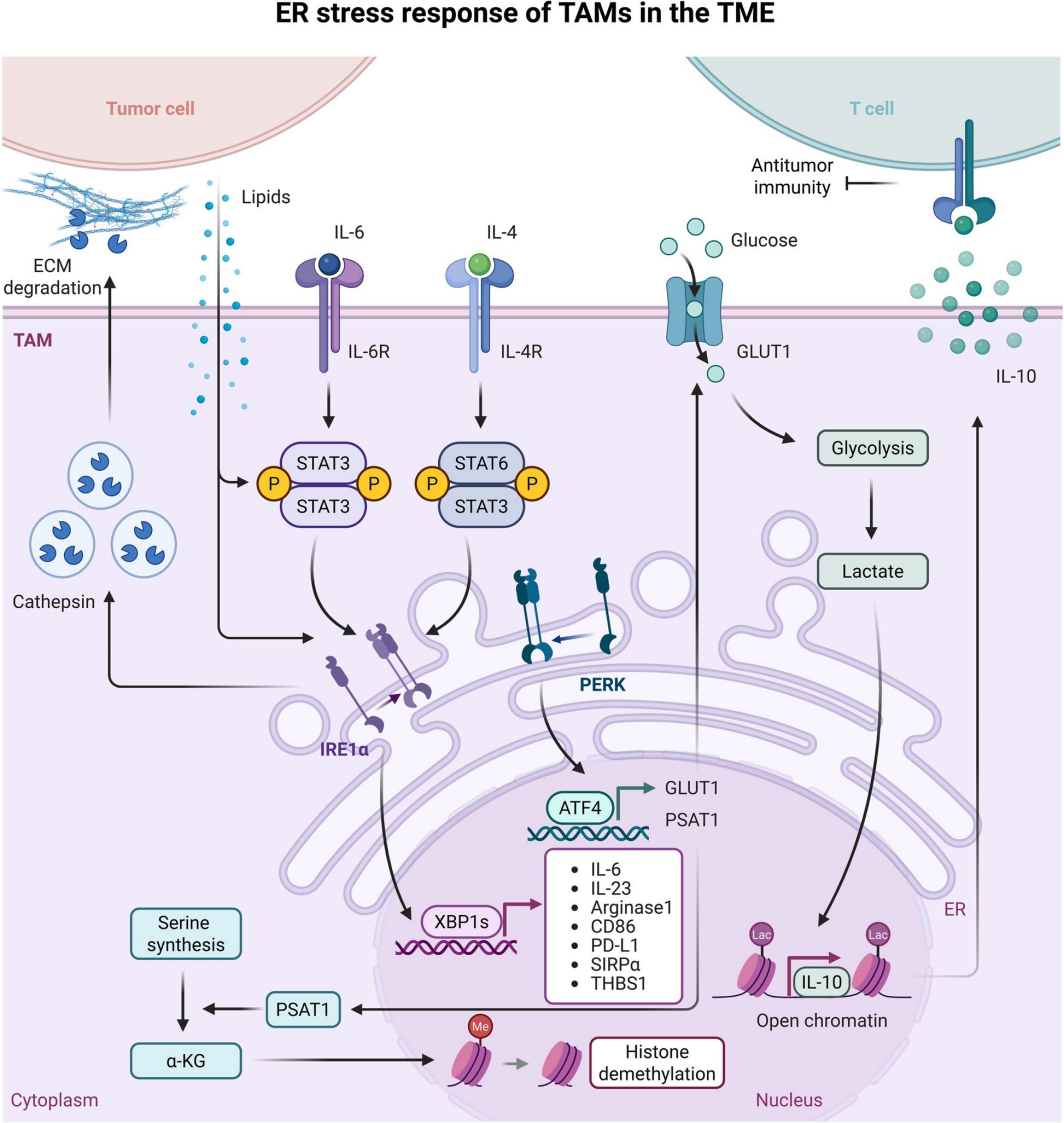
ER stress response of TAMs in the TME. The activation of ER stress in TAMs facilitates their polarization to a pro-tumorigenic and immunosuppressive phenotype. The IRE1α-XBP1 signaling induces macrophage polarization by upregulating IL-6, IL-23, Arginase1, CD86, and PD-L1. Cytokines (e.g., IL-4 and IL-6) activate the IRE1α signaling by activating the STAT6 and STAT3 pathways, thereby facilitating cathepsin secretion, ECM degradation, and tumor progression. Tumor cell-derived lipids induce ER stress in TAMs and thus sustain their survival and pro-tumor activity by activating the IRE1α-XBP1 and STAT3 pathways. The PERK signaling is also activated in TAMs and promotes M2 phenotype. The activation of this signaling facilitates mitochondrial respiration and upregulates PSAT1 and intrinsic serine biosynthesis via ATF4. PSAT1-induced serine synthesis balances the generation of α-KG required for JMJD3 histone demethylation and enhances M2 phenotype. PERK also upregulates GLUT1 expression and facilitates glucose metabolism in immunosuppressive TAMs depending on glucose-driven histone lactylation. Abbreviations: ER, endoplasmic reticulum; TAMs, tumor-associated macrophages; TME, tumor microenvironment; IRE1α, inositol-requiring enzyme 1α; PERK, protein kinase R-like ER kinase; ATF4, activating transcription factor 4; XBP1s, spliced form of X-box-binding protein 1; IL, interleukin; PD-L1, programmed death ligand 1; PSAT1, phosphoserine aminotransferase 1; α-KG, α-ketoglutarate; GLUT1, glucose transporter type 1

Macrophages are an essential component of the TME [132, 133]. These macrophages undergo ER stress activation and polarization to a pro-tumorigenic and immunosuppressive phenotype, facilitating immune dysregulation and failure of local immunosurveillance (Fig. 4) [20].

The IRE1α-XBP1 signaling induces macrophage polarization by upregulating IL-6, IL-23, Arginase1, CD86, and PD-L1 [134]. Loss of IRE1α in macrophages can prolong survival of tumor-bearing mice [134]. In addition to inhibition of the pro-tumor cytokines, loss of XBP1 also downregulates the expression of SIRPα and THBS1 in TAMs, thereby preventing “don’t eat me” signaling and enhancing phagocytosis [19]. Cytokines (e.g., IL-4/−6) elicit the IRE1α signaling in macrophages by activating the STAT6 and STAT3 pathways, thereby promoting cathepsin secretion and contributing to tumor progression [135]. Furthermore, tumor cell-derived lipids elicit ER stress to sustain macrophage survival and pro-tumor activity by activating the IRE1α-mediated XBP1 mRNA splicing and STAT3 pathway [115].

The PERK signaling is activated in TAMs, which drives immunotherapy resistance in lymph node metastases of OSCC [136]. The PERK signaling activation facilitates immunosuppressive M2 phenotype [137], and suppression of this signaling polarizes TAMs towards M1 phenotype [21] and enhances anti-PD-1 therapy in lymph node metastases of OSCC [136]. The PERK signaling activation also facilitates mitochondrial respiration to meet cellular energy demands, as well as upregulates phosphoserine aminotransferase 1 (PSAT1) and intrinsic serine biosynthesis through ATF4 [137]. In addition to promoting mitochondrial fitness, PSAT1-induced serine synthesis balances the generation of α-ketoglutarate (α-KG) required for JMJD3 histone demethylation and potentiates M2 macrophage phenotype. PERK also supports glucose transporter type 1 (GLUT1) upregulation and glucose metabolism in immunosuppressive TAMs in glioblastoma (GBM) based on glucose-driven histone lactylation [22]. Genetic loss of PERK in TAMs promotes CD8^+^ T cell infiltration in tumors, as well as improves the efficacy of anti-4-1BB immunotherapy [22]. Altogether, the PERK signaling acts as a key regulator for immunosuppressive TAMs.

Tumor cells persistently endure ER stress and can pass this stress on to adjacent cells and the TME. Transmissible ER stress is a key regulator of intercellular communications within the TME. Both tumor cells and macrophages can sense ER stress [138]. ER-stressed tumor cells also transmit ER stress to macrophages, enabling the direction of macrophages towards M2 phenotype and contributing to tumor progression [139]. Tumor cells also sense transmissible ER stress from M1 and M2 macrophages with distinct extent of ER stress activation [138]: M1 macrophages experience less ER stress but transfer more ER stress to tumor cells, accompanied with much release of proinflammatory cytokines, chemokines (e.g., IL-1β/−2/−6 and TNFα), and DAMPs; in contrast, M2 macrophages sense more but transmit less ER stress to tumor cells, accompanied with the release of anti-inflammatory molecules and less DAMPs. Furthermore, tumor cells and M2 macrophages survive upon ER stress, thereby promoting tumor growth. Therefore, understanding transmissible ER stress between tumor cells and the TME components, especially macrophages, is crucial for enhancing immunotherapy efficacy.

#### MDSCs

**Fig. 5:**
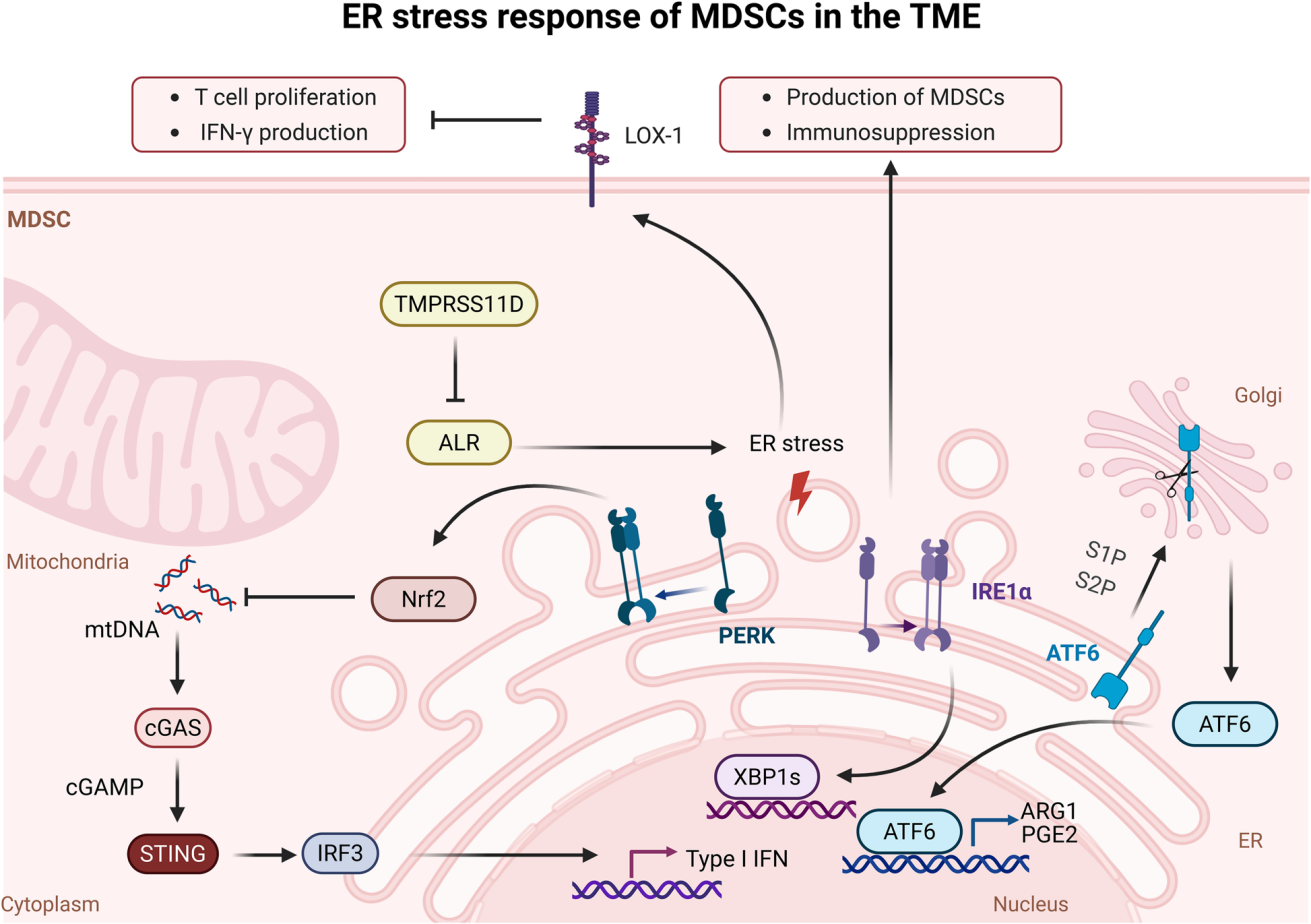
ER stress response of MDSCs in the TME. The activation of ER stress supports the immunosuppressive function of MDSCs. The IRE1α signaling is crucial for MDSC production and immunosuppression. The PERK signaling is activated in MDSCs, and the deficiency of this signaling converts MDSCs to myeloid cells that enhance antitumor immunity of CD8^+^ T cells. This effect is achieved by inhibiting the Nrf2 signaling, which triggers STING-dependent generation of type I IFN by accumulating cytosolic mtDNA. ER stress induces the production of LOX-1^+^ PMN-MDSCs and thus inhibits T cell proliferation and IFN-γ production. TMPRSS11D promotes ER stress in PMN-MDSCs by negatively regulating ALR, thereby improving the immunosuppression of PMN-MDSCs. Abbreviations: ER, endoplasmic reticulum; MDSCs, myeloid-derived suppressor cells; TME, tumor microenvironment; IRE1α, inositol-requiring enzyme 1α; PERK, protein kinase R-like ER kinase; ATF6, activating transcription factor 6; XBP1s, spliced form of X-box-binding protein 1; IFN, interferon; mtDNA, mitochondrial DNA; PMN-MDSCs, polymorphonuclear MDSCs; TMPRSS11D, transmembrane serine protease 11D; ALR, augmenter of liver regeneration

MDSCs are a heterogeneous myeloid cell population and accumulate in the TME where they suppress antitumor immunity of T and NK cells and exert tumor-promoting effects [140]. MDSCs also induce resistance to cancer treatments, especially immunotherapy [141, 142]. ER stress is activated in MDSCs [143], which enhances their immunosuppressive function (Fig. 5) [26].

The IRE1α signaling acts as a crucial regulator for MDSCs, and inhibition of this signaling abrogates production and immunosuppression of MDSCs [27]. The PERK signaling is also activated in MDSCs in the TME, and inhibition of this signaling converts MDSCs to myeloid cells, enabling the activation of CD8^+^ T cell-mediated antitumor immunity [26]. This effect is achieved by inhibiting the Nrf2 pathway, which triggers STING-mediated type I IFN by accumulating cytosolic mitochondrial DNA (mtDNA). Hence, inhibition of ER stress sensors can transform tumor-associated MDSCs into T cell-stimulating cells, achieving the synergistic antitumor efficacy when combining with anti-PD-L1 antibody and adoptive T cell transfer (ACT) in melanoma and lymphoma mouse models [26].

MDSCs can be classified into monocytic MDSCs (M-MDSCs) and polymorphonuclear MDSCs (PMN-MDSCs), which share phenotypic and morphologic characteristics with monocytes and neutrophils, respectively [144]. The immunosuppressive activity of PMN-MDSCs in tumors is driven by the IRE1α and ATF6 sensors [144]. ER stress induces lectin-type oxidized LDL receptor-1 (LOX-1)^+^ PMN-MDSCs, inhibiting proliferation and IFN-γ secretion in T cells [145]. Transmembrane serine protease 11D (TMPRSS11D) induces ER stress of PMN-MDSCs via negative modulation of augmenter of liver regeneration (ALR) and thus enhances the immunosuppression of PMN-MDSCs on T cells [146]. Oppositely, ER stress may be dispensable for the immunosuppression of M-MDSCs in tumors [144]. High IL-6 levels in the TME support the immunosuppression of M-MDSCs [144].

#### Neutrophils

**Fig. 6:**
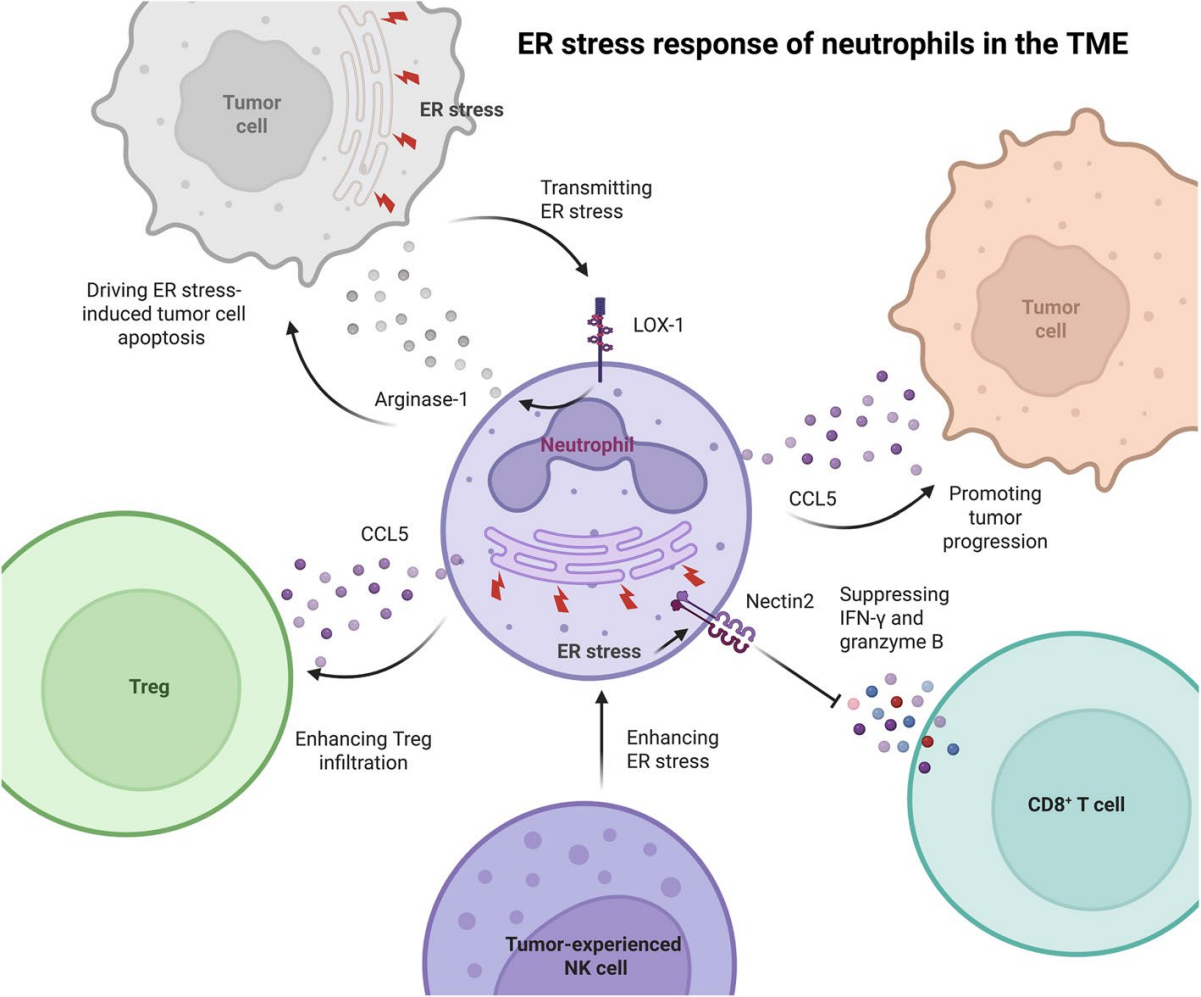
ER stress response of neutrophils in the TME. The activation of ER stress drives the polarization of neutrophils towards a pro-tumor phenotype. The pro-tumor neutrophils increase the secretion of CCL5 and thus promote tumor progression and enhance the infiltration of Tregs in the TME. ER stress upregulates the expression of Nectin2 in neutrophils, which directly impedes the secretion of IFN-γ and granzyme B by CD8^+^ T cells. Tumor-experienced NK cells enhance ER stress in neutrophils and sustain their ability to suppress antitumor immunity of CD8^+^ T cells. ER stress of tumor cells can be transmitted to neutrophils and converts them into a LOX-1.^+^ immunosuppressive phenotype. Neutrophils release arginase-1 to drive ER stress-induced apoptosis of tumor cells. Abbreviations: ER, endoplasmic reticulum; TME, tumor microenvironment; NK, natural killer; Treg, regulatory T cell; IFN, interferon; LOX-1, lectin-like oxidized low-density lipoprotein receptor-1; CCL5, C–C motif ligand 5

Neutrophils are implicated in tumor progression and immune escape [147]. Emerging evidence suggests the involvement of neutrophils in cancer immunotherapy resistance [148, 149]. ER stress response is elevated in tumor-infiltrating neutrophils [24], driving neutrophil polarization towards a pro-tumor phenotype [150, 151] (Fig. 6). The pro-tumor neutrophils increase the secretion of CCL5, facilitating tumor progression and enhancing Treg infiltration in the TME [150, 151].

ER stress upregulates Nectin2 expression in neutrophils, which directly impedes IFN-γ and granzyme B secretion by CD8^+^ T cells [150]. In addition to tumor cells [150, 151], tumor-experienced NK cells also enhance ER stress in neutrophils and sustain their ability to suppress antitumor immunity of CD8^+^ T cells [152]. Neutrophil-intrinsic IRE1α drives early adaptive immune escape in high-grade serious ovarian cancer, and selective loss of IRE1α in neutrophils can sensitize tumors to anti-PD-1 therapy [24]. Accordingly, targeting the ER stress sensors in neutrophils may overcome immunotherapy resistance.

In the peripheral blood of healthy subjects, LOX-1 is usually undetectable in neutrophils [23]. In contrast, in cancer patients, 5–15% of total neutrophils and 15–50% of neutrophils in tumors are LOX-1 positive. ER stress in tumor cells can be transmitted to neutrophils and converts them into LOX-1^+^ immunosuppressive phenotype [153]. Neutrophils release arginase-1 to drive ER stress-induced apoptosis of tumor cells [154]. LOX-1^+^ neutrophils are characterized by immunosuppressive function and ER stress activation of PMN-MDSCs [23]. Inducing ER stress in healthy neutrophils upregulates LOX-1 and thus converts them to PMN-MDSCs. Therefore, LOX-1 may be a biomarker for defining the subpopulation of PMN-MDSCs among neutrophils.

#### Dendritic cells (DCs)

DCs are potent antigen-presenting cells that orchestrate the interface between innate and adaptive immunity. They present tumor antigens to prime and activate T cells and thus drive antitumor immune responses [155]. ICIs cannot elicit potent immune responses in patients with limited and impaired DCs in the TME [156]. DCs show the constitutive activation of the IRE1α-XBP1 signaling, without canonical ER stress [157]. PGE2 released by tumor cells is one of the factors that transfer ER stress from tumor cells to DCs, causing their dysfunction [158]. To develop more effective cancer immunotherapy, it is crucial to understand the role of ER stress in modulating DCs’ phenotype and function.

**Fig. 7:**
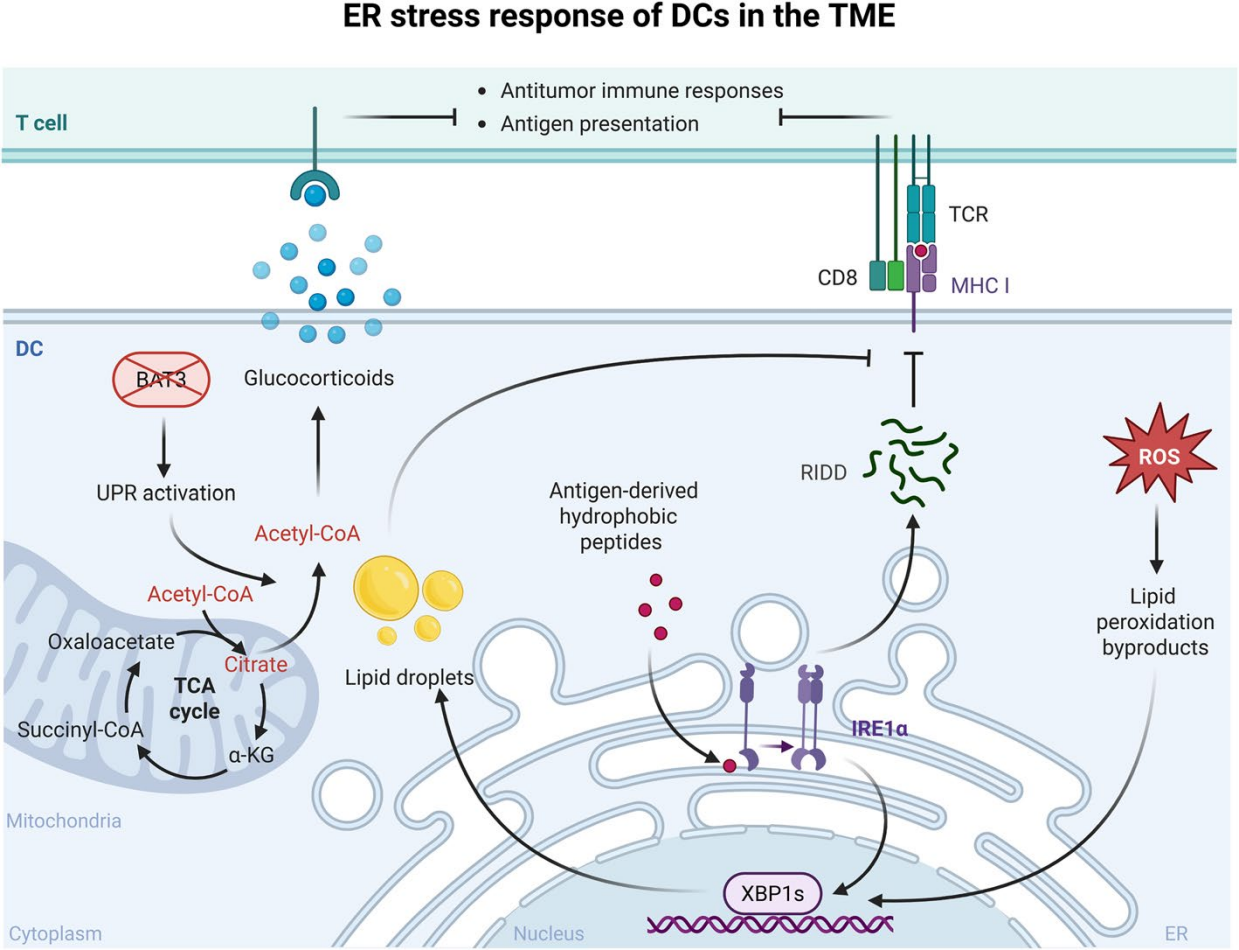
ER stress response of DCs in the TME. ER stress is activated in tumor-associated DCs within the TME. Increased ROS promotes the generation of lipid peroxidation byproducts, which trigger ER stress and XBP1 activation. The sustained activation of XBP1 destroys intracellular lipid homeostasis and induces lipid droplet accumulation, thereby impairing antigen presentation to T cells. The activation of IRE1α depletes MHC-I heavy-chain mRNAs via the RIDD mechanism and thus inhibits antigen cross-presentation of DCs. The deficiency of BAT3 activates ER stress in DCs and redirects acetyl-coenzyme A to enhance cell intrinsic steroidogenesis, which subsequently impairs antitumor immunity of T cells. Abbreviations: ER, endoplasmic reticulum; DCs, dendritic cells; TME, tumor microenvironment; IRE1α, inositol-requiring enzyme 1α; RIDD, regulated IRE1-dependent decay; XBP1s, spliced form of X-box-binding protein 1; MHC-I, MHC class I; ROS, reactive oxygen species; UPR, unfolded protein response; BAT3, HLA-B associated transcript-3

Tumor-associated DCs in the TME display upregulated ER stress markers and sustained XBP1 activation than non-cancerous DCs (Fig. 7) [11]. Furthermore, these DCs show elevated levels of ROS, promoting the generation of lipid peroxidation byproducts (e.g., 4-hydroxy-trans-2-nonenal (4-HNE)) that trigger ER stress and XBP1 activation [11]. The sustained activation of XBP1 destroys intracellular lipid homeostasis and induces accumulation of lipid droplets, thereby impairing antigen presentation to T cells [11].

Cross-presentation of antigens by DCs is important for eliciting effective antitumor immune responses [159]. XBP1 knockout alters lipid metabolism and improves cross-presentation of tumor antigens and antitumor immunity [11]. Antigen-derived hydrophobic peptides directly bind to IRE1α and masquerade as unfolded proteins [34]. The activated IRE1α consumes MHC-I heavy-chain mRNAs via the RIDD mechanism and thus curtails antigen cross-presentation. The ER chaperone HLA-B associated transcript-3 (BAT3) exhibits decreased expression in DCs from MC38 tumor-bearing mice than those from naive mice [28]. The deficiency of BAT3 results in UPR activation in DCs and redirects acetyl-coenzyme A to enhance cell intrinsic steroidogenesis, thereby impairing antitumor immune responses. Collectively, these studies suggest that inhibition of the IRE1α-XBP1 signaling in DCs may enhance antitumor immune responses.

### Lymphocyte dysfunction

#### T cells

**Fig. 8:**
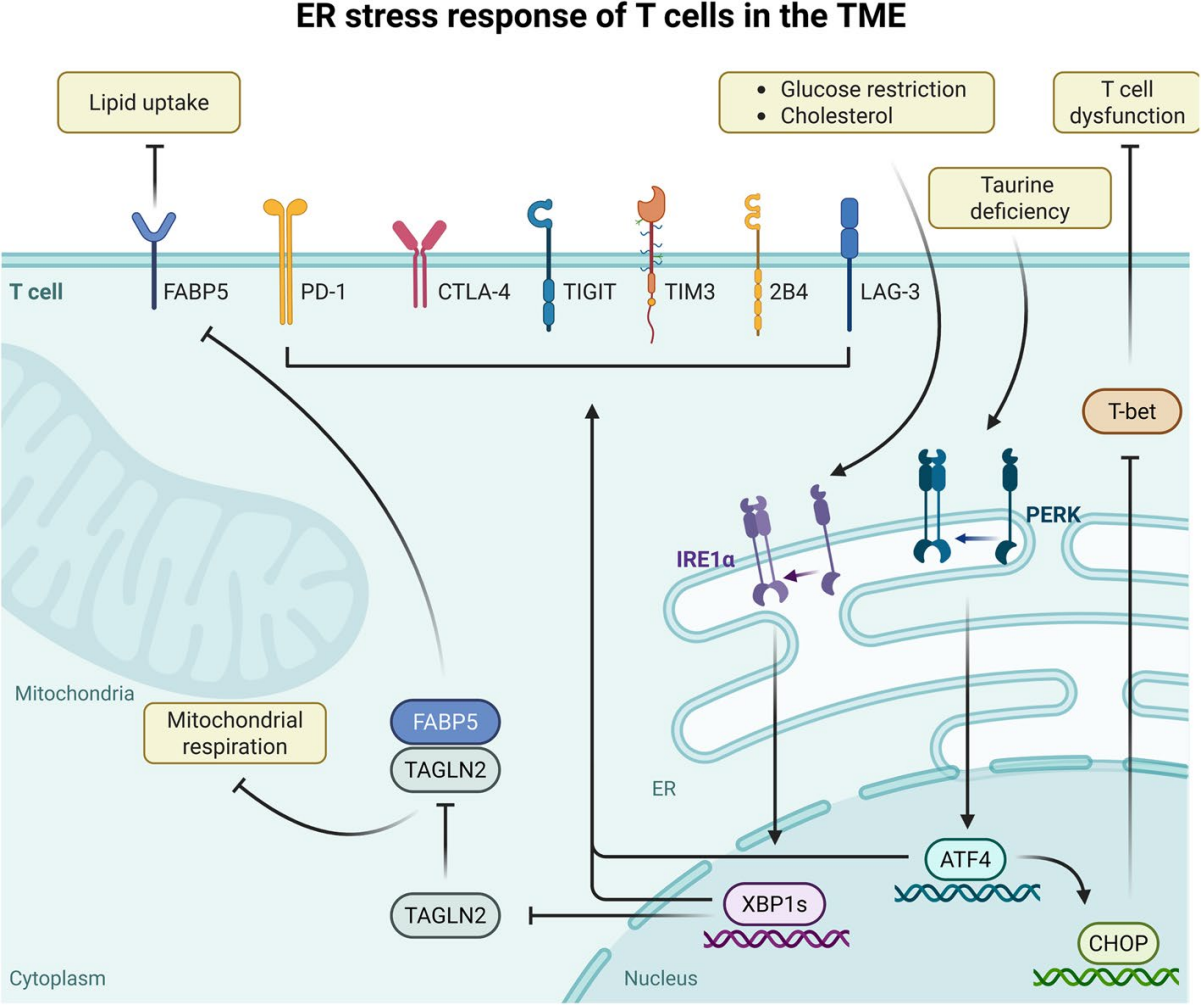
ER stress response of T cells in the TME. The IRE1α-XBP1 signaling destroys T cell metabolism and antitumor immunity. Cholesterol in the TME elicits CD8^+^ T-cell exhaustion and upregulates immune checkpoints (e.g., PD-1, 2B4, TIM3, and LAG-3) depending on ER stress. ER stress-induced XBP1s transcriptionally represses TAGLN2 and thus induces dysfunction of CD8^+^ T cells. Restricted glucose availability activates the IRE1α-XBP1 signaling. The PERK signaling induces chronic ER stress in CD8^+^ T effector cells and causes their mitochondrial exhaustion. CHOP is a downstream sensor of ER stress and upregulated in tumor-infiltrating CD8^+^ T cells mainly via the PERK/ATF4 signaling. CHOP induces CD8^+^ T cell dysfunction by repressing the expression of T-bet, a major regulator of effector T cell function. Taurine deficiency in CD8^+^ T cells enhances ER stress by inducing ATF4 transcription based on the PERK-JAK1-STAT3 axis. The increase in ATF4 expression transactivates many immune checkpoints (e.g., PD-1, CTLA-4, TIGIT, and TIM3) and elicits T cell exhaustion. Abbreviations: ER, endoplasmic reticulum; TME, tumor microenvironment; IRE1α, inositol-requiring enzyme 1α; PERK, protein kinase R-like ER kinase; ATF4, activating transcription factor 4; XBP1s, spliced form of X-box-binding protein 1; CHOP, C/EBP homologous protein; TAGLN2, Transgelin 2; PD-1, programmed cell death protein-1; 2B4, CD244; TIM3, T cell immunoglobulin and mucin domain-3; LAG-3, lymphocyte-activation gene-3; CTLA-4, cytotoxic T-lymphocyte-associated antigen-4; TIGIT, T cell immunoglobulin and ITIM domain

CD8^+^ T cells are the end effectors of antitumor immunity, and effective immunotherapy relies on the effector functions of CD8^+^ T cells [160]. The IRE1α-XBP1 signaling is a central orchestrator of tumor progression and immunosuppression in many cancer types, which is leveraged to destroy T cell metabolism and antitumor immunity (Fig. 8) [29].

Cholesterol in the TME elicits CD8^+^ T cell exhaustion [31] and upregulates immune checkpoints (e.g., PD-1, 2B4, TIM3, and LAG-3) [46] by activating ER stress. Transgelin 2 (TAGLN2) is required for fatty acid uptake, mitochondrial respiration, and antitumor function in CD8^+^ T cells by interacting with FABP5 to promote its cell surface localization [30]. ER stress-induced XBP1s transcriptionally represses TAGLN2, causing CD8^+^ T cell dysfunction. Clinically, upregulation of XBP1 is linked to decreased T cell accumulation in the TME [29].

Malignant ascites in patients with ovarian cancer suppresses glucose uptake and causes defects in N-linked glycosylation in T cells, activating the IRE1α-XBP1 signaling and suppressing mitochondrial activity and IFN-γ production [29]. Although the UPR usually promotes ER stress adaptation under adequate nutrition, T cells exhibit maladaptive IRE1α-XBP1 activation in the TME with restricted glucose availability. In multiple myeloma, inhibition of IRE1α decreases mitochondrial ROS (mtROS) levels in bone marrow-derived CD8^+^ T cells, improving antitumor immunity [161].

The PERK signaling induces chronic ER stress in CD8^+^ T effector cells and causes their mitochondrial exhaustion [162]. CHOP is a downstream sensor of ER stress and upregulated in CD8^+^ T cells in the TME mainly via the PERK/ATF4 signaling [163]. CHOP elicits CD8^+^ T cell dysfunction by repressing the expression of T-bet, a major effector function regulator of T cells. CHOP deficiency in T cells improves antitumor immunity and immunotherapy efficacy.

Taurine restriction in CD8^+^ T cells enhances ER stress by transcriptionally activating ATF4 based on the PERK-JAK1-STAT3 axis [106]. The upregulated ATF4 transactivates many immune checkpoint genes (PD-1, CTLA-4, TIGIT, and TIM3) and elicits T cell exhaustion. Supplementation of taurine can improve CD8^+^ T cell function and enhance the efficacy of anti-PD-1 antibody in both melanoma and breast cancer mouse models [164]. Therefore, taurine enhances antitumor immunity of T cells and overcomes T cell dysfunction caused by ER stress. Targeting the ER stress sensors offers a potential strategy for improving immunotherapy efficacy.

#### NK cells

**Fig. 9:**
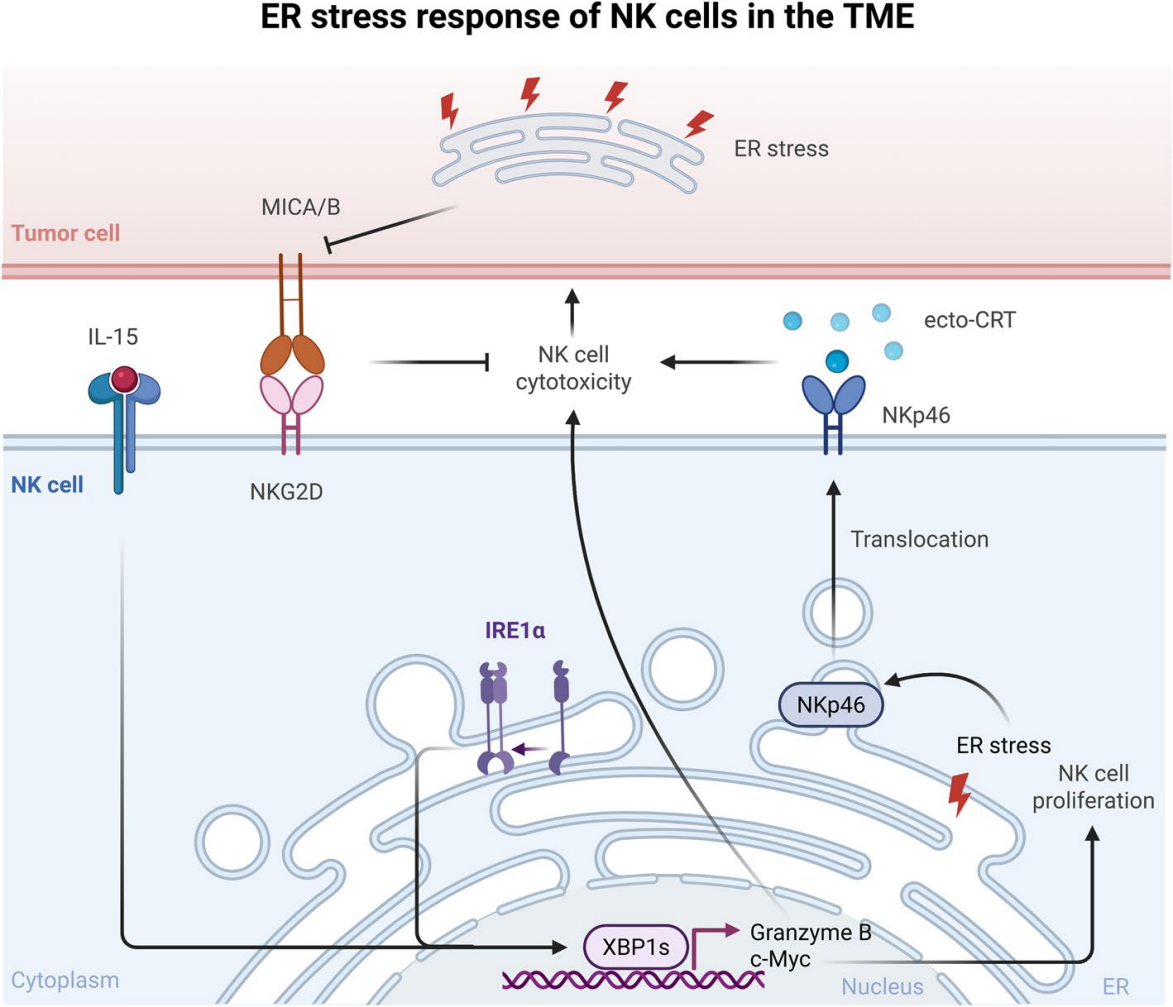
ER stress response of NK cells in the TME. MICA/B, major ligands for NKG2D, are inhibited by ER stress sensors. The downregulation of MICA/B impairs the sensitivity of tumor cells to NK cell cytotoxicity. In response to ER stress, NKp46 recognizes ecto-CRT and translocates from the ER to the cell surface, thereby eliminating ER-stressed cells. XBP1s is activated upon IL-15, which binds to and recruits the transcription factor T-BET to granzyme B, leading to transcriptional activation. c-Myc is a direct downstream target of XBP1 for regulating NK cell proliferation. XBP1s positively regulate the cytolytic activity of NK cells against leukemia cells, which is essential for IL-15-mediated NK cell survival based on an anti-apoptotic mechanism. Abbreviations: ER, endoplasmic reticulum; NK, natural killer; TME, tumor microenvironment; IRE1α, inositol-requiring enzyme 1α; XBP1s, spliced form of X-box-binding protein 1; IL, interleukin; MICA/B, major histocompatibility complex class I polypeptide-related sequence A/B; NKG2D, natural killer group 2 D; ecto-CRT, externalized calreticulin

NK cells are key effector cells for tumor cell killing via direct cytolytic activity or immunomodulatory signals to T cells and antigen presenting cells [165]. NK cells are associated with enhanced tumor control effects in various cancer types and better patient prognosis. However, NK cells’ function is usually impaired in the TME, with ER stress as a key factor (Fig. 9).

Major histocompatibility complex class I polypeptide-related sequence A/B (MICA/B), main ligands for natural killer group 2 D (NKG2D), are inhibited by the UPR signaling [166, 167]. The downregulation of MICA/B attenuates the sensitivity of hepatocellular carcinoma (HCC) to NK cell cytotoxicity [167].

Most NK cells express the NK cell receptor NKp46, and blocking it suppresses tumor killing of NK cells [32]. ER stress induces recognition of externalized calreticulin (ecto-CRT) by NKp46 and ER-cell surface translocation, thereby eliminating ER-stressed cells [168]. NKp46 recognition of ecto-CRT prevents tumor growth of melanoma and lung cancer, as well as enhances the degranulation of tumor-infiltrating NK cells and the secretion of cytokines.

The IRE1α-XBP1 signaling is intrinsically essential for expansion of activated NK cells and drives NK cell-mediated antitumor immunity, with c-Myc being a XBP1’s downstream target for modulating NK cell proliferation [45]. XBP1s is activated in response to IL-15, which induces T-BET-mediated transcriptional activation of granzyme B [169]. XBP1s positively modulates the cytotoxicity of NK cells against leukemia cells, which is essential for IL-15-driven NK cell survival [169].

Altogether, unleashing NK cell activity by ER stress holds great potential to improve cancer immunotherapy [165].

#### B cells

**Fig. 10:**
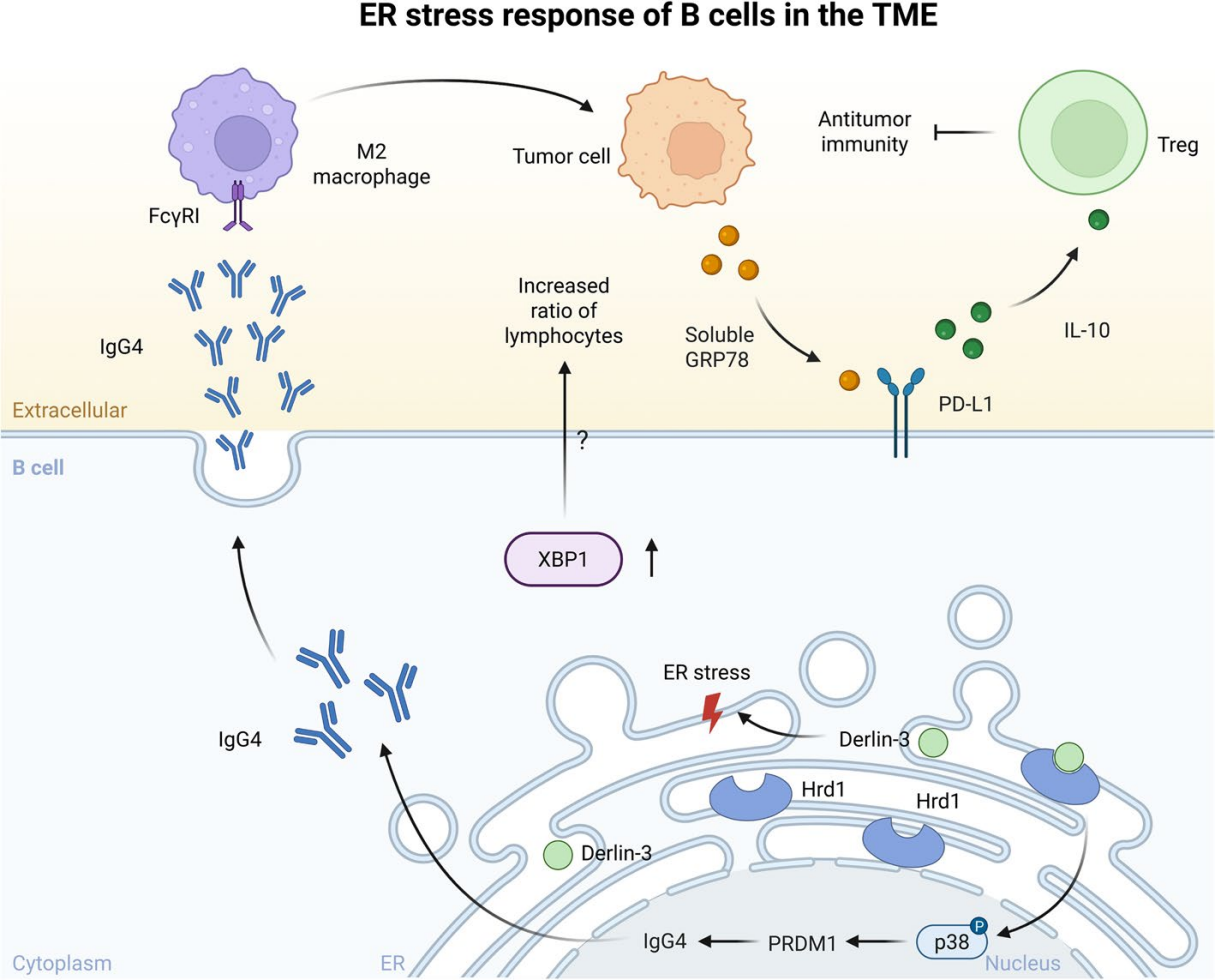
ER stress response of B cells in the TME. An increase in XBP1 expression in plasma cells is linked to an increase in the ratio of lymphocytes, indicating that XBP1-mediated ER stress may modulate the crosstalk between optimal acquired humoral immune response and innate immunity. Soluble GRP78 binds with tumor-infiltrating B cells and converts them into IL-10^+^/PD-L1^+^ B cells, thereby facilitating the formation of Tregs and inhibiting antitumor immunity of T cells. Derlin-3 manipulates ER stress and IgG4 secretion in plasma cells via targeting the Hrd1/p38/PRDM1 signaling. Abbreviations: ER, endoplasmic reticulum; TME, tumor microenvironment; XBP1s, X-box-binding protein 1; GRP78, glucose‐regulated protein 78; PD-L1, programmed death ligand 1; IL, interleukin; Treg, regulatory T cell; IgG, immunoglobins G

B cells are an essential component of the TME, which promote immune response by antigen presentation, cytokine secretion, and differentiation into antibody-secreting plasma cells [170]. B cells are associated with cancer patient outcomes and immunotherapy response [171–173]. ER stress regulates B cell-mediated immune response (Fig. 10).

High XBP1 expression in plasma cells indicates an undesirable prognosis of esophageal cancer patients [174]. Nevertheless, XBP1 upregulation in plasma cells is linked to an increase in the ratio of lymphocytes, indicating that XBP1-mediated ER stress may modulate the crosstalk between innate and acquired immune responses [174].

Soluble GRP78 binds with tumor-infiltrating B cells and converts them into IL-10^+^/PD-L1^+^ B cells, facilitating the generation of Tregs, as well as inhibiting the antitumor immunity of T cells [25]. Accordingly, targeted degradation of soluble GRP78 can effectively reverse the immunosuppressive TME.

Derlin-3 acts as a key component in the degradation of misfolded lumen glycoproteins elicited by ER stress [175, 176]. Derlin-3 is primarily expressed in B cells in the lung adenocarcinoma (LUAD) TME, especially in plasma cells [177]. It manipulates ER stress and immunoglobins G (IgG)4 secretion in plasma cells through modulating the Hrd1/p38/PRDM1 signaling [177]. Inhibition of Derlin-3 reduces plasma cell accumulation and M2 macrophage polarization, indicating Derlin-3-mediated ER stress as an immunotherapeutic target in LUAD.

## Development of therapeutic strategies and biomarkers of ER stress in cancer immunotherapy

Abnormal activation of ER stress sensors and their effectors are crucial regulators of response and resistance to cancer immunotherapy. Therapeutic strategies targeting ER stress improve immunotherapy efficacy and overcome resistance. Moreover, these molecules hold potential as biomarkers for predicting immunotherapy outcomes.

### Therapeutic strategies targeting ER stress sensors for enhancing cancer immunotherapy and overcoming resistance

Targeting ER stress sensors holds potential for improving immunotherapy efficacy and overcoming resistance, especially in combination with ICIs (Table 1).

**Table 1:** Targeting ER stress signaling to enhance the efficacy of cancer immunotherapy and overcome resistance

Compounds	Combined treatment	Cancer type	Effect	References
Targeting IRE1α signaling				
4μ8C	Chemotherapy + anti-PD-1 antibody	Cervical cancer	Improving the efficacy of chemoimmunotherapy and overcoming resistance	[178]
MKC8866	Anti-PD-1 antibody	Prostate cancer	Enhancing the efficacy of anti-PD-1 therapy and reprogramming the TME (e.g., boosting the infiltration of CD8^+^ T and NK cells)	[18]
	Taxane + anti-PD-1 antibody	TNBC	Converting PD-L1-negative, ICI-unresponsive tumors into PD-L1^high^ immunogenic tumors hyper-sensitive to ICIs	[179]
G9668	Anti-PD-L1 antibody	TNBC	Causing tumor regression and increasing the infiltration and activation of DCs and CD8^+^ T cells	[34]
B-I09	Anti-PD-1 antibody	CARM1-expressing ovarian cancer	Synergistically inhibiting tumor burden and prolonging the survival time	[35]
KIRA6	Anti-PD-1 antibody	Breast cancer	Diminishing MDSC production, restoring T cells, and overcoming resistance to anti-PD-1 therapy	[27]
Targeting PERK signaling				
GSK2656157	Anti-PD-1 antibody	Melanoma	Enhancing the efficacy of anti-PD-1 therapy	[137]
	Erastin + anti-CTLA-4 antibody	Pancreatic cancer with cTRIP12 overexpression	Showing an effective antitumor efficacy	[180]
GSK2606414	Anti-PD-1 antibody	Sarcoma	Enhancing the efficacy of anti-PD-1 therapy and prolonging the survival time	[162]
Targeting GRP78				
HA15	Anti-PD-1 antibody	Melanoma	Enhancing CD8^+^ T cell-dependent antitumor immunity and potentiating the efficacy of immunotherapy	[33]
*Clostridium butyricum*	Anti-PD-1 antibody	CRC	Improving anti-PD-1 efficacy	[181]
Celastrol-loaded ginsenoside Rg3 liposome	Anti-PD-L1 antibody	TNBC	Provoking ICD and ER stress, activating DCs and boosting antigen presentation, and improving treatment efficacy	[182]
CAR T cells targeting GRP78				
GRP78-CAR T cells	-	Solid and brain tumors	Recognizing and killing GRP78^+^ tumors	[183–185]
	-	AML	Showing robust anti-AML activity, without targeting hematopoietic progenitor cells	[186, 187]
	Gemcitabine	Pancreatic cancer	Specifically killing tumor cells and gemcitabine-resistant cells	[188]
GRP78- and CD123-bispecific CAR T cells	-	AML	Conferring CAR T cell multiantigen specificity and preventing immune escape	[189]

*Abbreviations*: *IRE1α* Inositol-requiring enzyme 1α, *PD-1* Programmed death 1, *TME* Tumor microenvironment, *NK* Natural killer, *PD-L1* Programmed death ligand 1, *ICI* Immune checkpoint inhibitors, *TNBC* Triple-negative breast cancer, *DCs* Dendritic cells, *CARM1* Coactivator-associated arginine methyltransferase 1, *MDSC* Myeloid-derived suppressor cell, *PERK* Protein kinase R-like ER kinase, *CTLA-4* Cytotoxic T-lymphocyte antigen 4, *cTRIP12* CircRNA derived from TRIP12, *eIF2α* Eukaryotic translation initiation factor 2 α, *TIGIT* T cell immunoreceptor with immunoglobulin and ITIM domain, *GRP78* Glucose‐regulated protein 78, *CRC* Colorectal cancer, *ICD* Immunogenic cell death, *ER* Endoplasmic reticulum, *CAR* Chimeric antigen receptor, *GBM* Glioblastoma, *IFN* Interferon, *AML* Acute myeloid leukemia

#### IRE1α signaling inhibitors

Targeting the IRE1α signaling by several IRE1α signaling inhibitors (e.g., MKC8866, 4μ8C, G9668, KIRA6, and B-I09) represents potential actionable strategies to improve immunotherapy efficacy.

The IRE1α RNase inhibitor MKC8866 (also called ORIN1001) monotherapy prevents tumor growth in several preclinical tumor models, e.g., prostate cancer [190] and soft-tissue sarcoma [191]. Furthermore, MKC8866 can enhance the response of anti-PD-1 antibody observed in prostate cancer mouse models [18]. The IRE1α RNase inhibitor MKC8866 is currently in phase I/II clinical assessment for treating advanced solid tumors (e.g., NCT05154201 and NCT03950570).

Chemotherapy is often in combination with ICIs to improve immunotherapy response, but many immunologically cold tumors are unresponsive. Chemotherapy selectively suppresses the translation of IRE1α [192]. Immunologically cold tumors hijack ER stress to suppress taxane chemotherapy-induced danger signals and impair antitumor immunity [179]. Furthermore, adaptive ER stress mediated by the IRE1α-XBP1 signaling in drug-tolerant persister (DTP) cells contributes to resistance to neoadjuvant chemoimmunotherapy [178]. The combination of MKC8866 with taxane can convert PD-L1^negative^ TNBC tumors into PD-L1^high^ tumors hypersensitive to ICIs [179]. In addition to MKC8866, pharmacological suppression of IRE1α by 4μ8C (a specific IRE1α inhibitor) in combination with chemoimmunotherapy (chemotherapy + anti-PD-1 antibody) can eradicate DTP cells in cervical cancer mouse models [178].

CARM1 serves as a co-activator of XBP1s to promote the expression of XBP1s targets [35]. Pharmacological suppression of the IRE1α/XBP1s signaling using B-I09 selectively inhibits CARM1^+^ ovarian cancer, which further synergizes with anti-PD-1 therapy [35]. Inhibition of IRE1α by G9668 (an inhibitor for IRE1α kinase and RNase activities) prevents IRE1α-mediated negative feedback that removes MHC-I heavy-chain mRNAs via RIDD to impair DC cross-presentation and reduce CD8^+^ T cell activation [34]. In TNBC mouse models, G9668 improves the response of anti-PD-L1 antibody, causing tumor regression [34].

KIRA6 is also an inhibitor for IRE1α kinase and RNase activities, which can diminish MDSC production and restore T cell infiltration, ultimately overcoming resistance to anti-PD-1 therapy [27]. However, this IRE1α inhibitor exhibits potential off-target effects [193]. Nanotechnology offers a promising platform for improving the bioavailability and stability of ER stress-targeted agents, reducing toxic side effects, and overcoming immunotherapy resistance [194, 195]. The KIRA6-loaded α-tocopherol nanoemulsion has been utilized for enhancing immunotherapy efficacy, including ICIs and DC vaccines. KIRA6 is encapsulated into a reductive nanoemulsion containing α-tocopherol, which has a dual effect on suppressing ER stress and oxidative stress of M2 TAMs [194]. In addition to delaying tumor growth, this nanoemulsion effectively improves the efficacy of anti-PD-1 antibody. Another nanoemulsion contains KIRA6, α-tocopherol, and anti-PD-1 antibody, which can convert the hepatic TME to a “hot” phenotype by repolarizing immunosuppressive hepatic macrophages, upregulating Th1-like effector CD4^+^ T cells, and rejuvenating DCs and CD8^+^ T cells [196].

Given the pivotal orchestrators of DCs in antitumor immunity, DC-based antitumor immunotherapies are being actively developed [197, 198]. DC activation induced by tumor cells and tumor antigens are traditional methods of DC vaccines [199]. Tumor cell-mediated DCs physiologically mimic tumor identification and rejection, causing DC-based immune recognition and migration towards the TME. Due to the existence of immunosuppressive factors in the TME, tumor cell-mediated DCs present hyperactivated oxidative stress and ER stress, causing their malfunction [200]. KIRA6-loaded α-tocopherol nanoemulsion can also ameliorate both oxidative stress and ER stress in tumor cell-mediated DCs, thereby improving the antitumor efficacy [200].

Collectively, future studies are needed to further develop and optimize IRE1α signaling inhibitors for clinical use.

#### PERK signaling inhibitors

PERK has also emerged as a promising treatment target in cancer. Several early generations of PERK inhibitors (e.g., GSK2656157 [201] and GSK2606414 [202]) exert anti-tumor effects. Furthermore, preclinical studies have uncovered the effect of PERK inhibitors in improving immunotherapy efficacy. In tumor-bearing mice of melanoma, GSK2656157 can delay tumor growth, suppress the immunosuppressive function of TAMs, and potentiate antitumor immunity of T cells [137]. Notably, GSK2656157 treatment potentiates the efficacy of anti-PD-1 immunotherapy against melanoma. The circRNA derived from TRIP12 (cTRIP12) upregulation causes ferroptosis resistance and immunosuppression in pancreatic cancer, which binds to PERK and thus increases the expression of ferritin heavy chain (FTH) and PD-L1 in tumor cells [180]. Based on this, the combined treatment containing GSK2656157, erastin, and anti-CTLA-4 antibody has been developed, showing an effective antitumor efficacy in cTRIP12-overexpressing tumors.

GSK2606414 abrogates mtROS in PD-1^+^CD8^+^ T cells and improves their viability [162]. Accordingly, GSK2606414 augments the efficacy of anti-PD-1 therapy in a sarcoma mouse model, with CD8^+^ T cells being essential for the combination therapy. Photodynamic therapy (PDT) elicits tumor immunogenic cell death (ICD) mainly by activating ER stress in tumor cells. Nevertheless, ER stress promotes the activation and proliferation of TAMs, which act as a key driver of immune escape [203]. To optimize PDT-based immunotherapy, it is crucial to coordinate the conflicting demands of ER stress signaling in tumor cells and TAMs. An adaptively transformable nanoplatform enables the delivery of a photosensitizer and the PERK signaling inhibitor GSK2606414 into tumor cells and TAMs, respectively, thereby enhancing photodynamic immunotherapy [203].

Despite this, these early generations of PERK inhibitors show notable problems in specificity and pancreatic toxicities [204, 205]. New generations of PERK inhibitors could show favorable selectivity and better safety [206]. The PERK inhibitors HC-5404-FU and NMS-03597812, are under phase I clinical trials in patients with advanced solid tumors (NCT04834778) and with relapsed or refractory multiple myeloma (NCT05027594), respectively.

#### ATF6 inhibitors

Several ATF6 inhibitors (e.g., Ceapin-A7 and AEBSF) have been developed. It has been demonstrated that Ceapin-A7 exerts anti-tumor effects, such as suppressing colorectal cancer (CRC) progression [207]; impairing the survival of castration-resistance prostate cancer cells [208]; and improving the radiotherapy sensitivity in pancreatic cancer [209]. However, it remains unclear whether ATF6 inhibitors enhance the efficacy of cancer immunotherapy and overcome resistance. This issue still deserves further investigation in future studies.

#### GRP78 inhibitors

GRP78 is overexpressed in many cancer cell types and associated with multiple oncogenous processes [82, 210]. Furthermore, GRP78 is predominantly expressed on the surface of cancer cells. Therefore, compounds enabling the direct inhibition of GRP78 in tumor cells hold promise for modulating ER stress and enhancing antitumor immunity.

HA15 is a specific inhibitor of ER chaperone GRP78 enabling the induction of ER stress by suppressing the ATPase activity of GRP78 [211]. HA15 enhances CD8^+^ T cell-mediated antitumor immunity in melanoma mouse models, which further potentiates the antitumor efficacy of anti-PD-1 antibody [33].

Except for small molecule compounds, probiotic *Clostridium butyricum* decreases GRP78 expression [181]. Surface protein secD of tumor-resident probiotic *Clostridium butyricum* binds to cell-surface GRP78 of CRC cells and thus induces GRP78 and PI3K-AKT-NF-κB inactivation, decreasing immunosuppressive IL-6 secretion [181]. *Clostridium butyricum* elicits CD8^+^ T cell activation and TAM impairment, notably in combination with anti-PD-1 antibody. More studies are required to determine whether the inactivation of GRP78 is essential for *Clostridium butyricum*-mediated antitumor immunity. The combination therapies of *Clostridium butyricum* and anti-PD-1 antibody are undergoing in phase I clinical trials in patients with urothelial carcinoma (NCT06696742) and with renal cell cancer after surgery (NCT07037004), respectively. Furthermore, the triple therapy of *Clostridium butyricum* and anti-PD-1 and anti-CTLA-4 antibodies is also being investigated in phase I clinical trials in patients with advanced kidney cancer (NCT03829111 and NCT06399419).

GRP78-targeted nanoplatforms have also been developed, especially in combination with ICIs. A GRP78-binding nanobody‐directed immunotoxin (C5‐PE38) has been developed, which inhibits tumor progression and metastasis through improving innate and adaptive immunity via activating the stimulator of interferon genes (STING) signaling [212]. The combination treatment of C5‐PE38 and anti‐PD-1 antibody improves antitumor immunity in primary and metastatic melanomas. Celastrol-loaded ginsenoside Rg3 liposome provokes ICD by downregulating GRP78 and eliciting ER stress in tumor cells [182]. Furthermore, this liposome activates DCs and enhances antigen presentation to T cells. Notably, its combination with anti-PD-L1 antibody enhances the treatment efficacy in TNBC.

#### Chimeric antigen receptor (CAR) T cells targeting GRP78

Adoptive cell transfer (ACT), e.g., CAR T cells, is a personalized cancer immunotherapy approach [213, 214]. Lack of targetable antigens represents a key limiting factor in successful T cell-based immunotherapy. The ER stress-associated molecules are promising immunotherapeutic targets since the ER stress modulates the capacity of tumor cells in resisting cell death and supporting proliferation and metastasis.

Given that GRP78 is upregulated and translocated to the cell surface of various tumor cell types upon ER stress, CAR T cells targeting cell-surface GRP78 (GRP78-CAR T cells) have been developed, enabling the recognition and killing of GRP78^+^ solid tumors [183–185]. In addition to solid tumors, GRP78-CAR T cells also exhibit effective anti-acute myeloid leukemia (AML) activity, without toxicity to hematopoietic progenitor cells [186, 187]. CAR T cells are safe and lead to sustained disease control in different tumor mouse models [184, 185, 187].

Although GRP78 presents overexpression on CAR T cells under T cell activation, its expression shows tumor cell specificity and leads to varying treatment responses [185]. These CAR T cells limit antitumor response in tumors with low to middle expression of GRP78, e.g., diffuse intrinsic pontine gliomas and Ewing sarcoma. RASA2 knockout can overcome the limited response of GRP78-CAR T cells when targeting tumors expressing low GRP78 levels [185].

Given that CAR T cells targeting a single antigen have a potential inherent risk of immune evasion, bispecific CAR T cells (78.123) have been developed, which target two antigens in a peptide- single-chain variable fragment (scFv) configuration, GRP78 and CD123 (IL-3 receptor α) [189]. The bispecific CAR T cells are capable of recognizing and killing AML cells that express GRP78 and/or CD123 on the cell surface, and enhance antitumor activity than their monospecific CAR T cells.

Due to the unique expression of GRP78 on the cell surface, it could become a key target for tumor-directed treatment. Further studies are required to translate these preclinical findings into clinical practice.

### ER stress molecules as prognostic and predictive biomarkers for cancer immunotherapy

The effectiveness of immunotherapy is confined to a subset of patients. Although PD-L1 expression by immunohistochemistry is the most often used predictive biomarker for immunotherapy, the efficacy is not ideal [215]. Meanwhile, tumor mutational burden (TMB) faces many technical problems as a predictive biomarker for immunotherapy [216]. It is urgently needed to identify additional biomarkers for predicting prognosis and monitoring the efficacy of cancer immunotherapy. At current, studies on ER stress have identified several prognostic and predictive biomarkers for cancer immunotherapy (Table 2).

**Table 2:** ER stress-related prognostic and predictive biomarkers for cancer immunotherapy

Biomarker	Cancer type	Mechanistic rationale	Association with immunotherapy response	Type	Current translational stage	Reference
XBP1s	Melanoma	Altered UPR signaling coincides with alterations of gut microbiota and antitumor immunity	XBP1s^low^ group vs. XBP1s^high^ group: median PFS: 555 days vs. 103 days, *p* = 0.021	Prognostic	Preclinical	[217]
ATF4	Melanoma	Altered UPR signaling coincides with alterations of gut microbiota and antitumor immunity	PFS: ATF4^low^ group > ATF4^high^ group, *p* = 0.0076	Prognostic	Preclinical	[217]
GRP78	Melanoma	Altered UPR signaling coincides with alterations of gut microbiota and antitumor immunity	GRP78^low^ group vs. GRP78^high^ group: median PFS: 23 months vs. 4 months, *p* = 0.021	Prognostic	Preclinical	[217]
PERK signaling mRNA score	Melanoma	PERK signaling in tumor cells restricts protective anti-tumor T cell immunity	PERK signaling^high^ group vs. PERK signaling^low^ group: OS: HR, 2.78; *p* < 0.01	Prognostic	Preclinical	[218]
ERO1A	Lung cancer	ERO1A in cancer cells induces transmissible ER stress in the TME, promotes an immunosuppressive TME, and impairs the response to anti-PD-1 therapy	ERO1A^low^ group vs. ERO1A^high^ group: i. Clinical response: *p* = 0.002; PR: 13 (86.7%) vs. 4 (18.2%); SD: 2 (13.3%) vs. 14 (63.6%); PD: 0 (0%) vs. 4 (18.2%) ii. RFS: HR, 0.26; 95% CI, 0.07–0.91; *p* = 0.034	Predictive/prognostic	Preclinical	[219]
HRS	Melanoma	Loss of HRS in tumor cells induces misfolded protein accumulation and ER stress, causing the activation of the type I interferon pathway via the IRE1α/XBP1 signaling	OS: HRS^low^ group > HRS^high^ group; In high TMB population: HRS expression: non-responder group > responder group	Predictive/prognostic	Preclinical	[220]

*Abbreviations*: *XBP1s* Spliced form of X-box-binding protein 1, *UPR* Unfolded protein response, *PFS* Progression-free survival, *ATF4* Activating transcription factor 4, *GRP78* Glucose‐regulated protein 78, *PERK* Protein kinase R-like ER kinase, *OS* Overall survival, *HR* Hazard ratio, *ERO1A* Endoplasmic reticular oxidoreductase-1α, *TME* Tumor microenvironment, *PD-1* Programmed death 1, *PR* Partial response, *SD* Stable disease, *PD* Progression of disease, *RFS* Relapse-free survival, *HRS* Hepatocyte growth factor-regulated tyrosine kinase substrate, *ER* Endoplasmic reticulum, *IRE1α* Inositol-requiring enzyme 1, *TMB* Tumor mutational burden

In melanoma patients receiving ICIs, the expression levels of XBP1s, ATF4, and GRP78 are lower in responders to ICIs compared with non-responders [217]. Furthermore, the low expression of XBP1s, ATF4, and GRP78 is associated with better progression-free survival (PFS) among these patients receiving ICIs [217]. In contrast, high PERK signaling mRNA score predicts worse overall survival (OS) in melanoma patients receiving anti-PD-1 and/or anti-CTLA-4 compared with those with low PERK signaling mRNA score [218]. Loss of hepatocyte growth factor-regulated tyrosine kinase substrate (HRS) causes misfolded protein accumulation and triggers ER stress, leading to type I interferon response in an IRE1α-XBP1-dependent mechanism [220]. Furthermore, loss of HRS sensitizes anti-PD-1 immunotherapy in melanoma mouse models. Clinically, high HRS expression is correlated with poor OS in patients with melanoma receiving anti-PD-1 immunotherapy [220]. For patients with high TMB, responders have a higher expression of HRS than non-responders.

In addition to predicting prognosis of patients receiving cancer immunotherapy, endoplasmic reticular oxidoreductase-1α (ERO1A) can be used to predict the response to immunotherapy [219]. ERO1A in cancer cells induces transmissible ER stress in the TME, which induces an immunosuppressive TME and impairs the response to anti-PD-1 therapy [219]. Tumor-intrinsic ERO1A deficiency enhances anti-tumor immunity and improves the efficacy of anti-PD-1 in tumor-bearing mice [219]. In patients with lung cancer, highly expressed ERO1A predicts a lower response to neoadjuvant immunotherapy and a higher recurrence risk after neoadjuvant immunotherapy [219]. These studies highlight the potential of using ER stress-related molecules as prognostic and predictive biomarkers for cancer immunotherapy. Detection of these biomarkers in patients could inform personalized immunotherapy strategies.

Although several single ER stress-related prognostic and predictive biomarkers for cancer immunotherapy have been identified, comparisons between single biomarkers and traditional biomarkers, such as PD-L1 expression status, remain limited. It is necessary to conduct a global evaluation on a variety of ER stress-related biomarkers, in order to improve the accuracy in predicting prognosis and immunotherapy response. Furthermore, the predictive performance of these biomarkers deserves further assessment in prospective cohorts, ideally in clinical trials.

## Conclusion

Although accumulating evidence suggests that therapeutic targeting of ER stress pathways can enhance antitumor immunity and immunotherapy efficacy, several key issues remain unresolved. In contrast to IRE1α/XBP1 and PERK, the research on ATF6 has been relatively lagging in the field, although it is also a major arm of the UPR. Future research should focus on the role and mechanism of ATF6 in antitumor immunity and immunotherapy resistance.

Many tumors show chronic ER stress activation, which may interfere with the efficacy of cancer immunotherapy. Understanding how specific ER stress sensors cause immunotherapy resistance may facilitate the development of novel therapeutic strategies for overcoming immunotherapy resistance and improving therapy efficacy. Although preclinical studies have demonstrated that pharmacological suppression of key ER stress sensors can reprogram the TME, improve antitumor immunity, and overcome immunotherapy resistance, the long-term and systemic effects require further investigation in clinical trials. The ongoing clinical trials investigating ER stress-targeted agents (e.g., NCT05154201, NCT03950570, NCT04834778, and NCT05027594) provide an opportunity to establish their application in cancer immunotherapy. However, further studies are required to determine the efficacy of ER stress-targeted agents in combination with immunotherapy, precise immunological consequences of therapeutic targeting ER stress, as well as to optimize strategies that utilize ER stress to improve antitumor immunity and minimize off-target effects. The interactions between ER stress in the TME and immune responses warrant in-depth exploration for successful integration of ER stress-targeted agents into immunotherapy. This will provide cancer patients with more effective and long-lasting treatment options.

It is important to clarify the specific influence of ER stress on immune cell functions in the TME. Single-cell and spatial multi-omics techniques could provide platforms to distinguish stress-adaptive and immunosuppressive pathways of ER stress. Understanding how ER stress regulation influences immune functions is crucial for rationally designing combined immunotherapy.

It is crucial to develop biomarkers enabling the prediction of patients who can benefit from therapies targeting ER stress. Because ER stress varies among distinct tumor types and patient subpopulations, biomarker-based patient stratification is of importance to ensure the accuracy of clinical practice. Determining ER stress biomarkers in tumor biopsies or circulating tumor/immune cells may offer an insight into treatment response prediction, facilitating personalized immunotherapy. Furthermore, future research should not only focus on the prognostic and predictive value of ER stress-related biomarkers for cancer immunotherapy but also on their potential role in immunotherapy resistance in prospective cohorts, especially in clinical trials.

Strategies targeting ER stress hold potential in cancer immunotherapy, which can remodel the TME, improve antitumor immunity, and combat immunotherapy resistance. With the increasingly deepened understanding of ER stress in immune modulation, more effective and durable next-generation cancer immunotherapies will be developed, thereby improving patient outcomes.

## Data Availability

No datasets were generated or analysed during the current study.
